# Potential population level impact on tuberculosis incidence of using an mRNA expression signature correlate-of-risk test to target tuberculosis preventive therapy

**DOI:** 10.1038/s41598-019-47645-z

**Published:** 2019-07-31

**Authors:** Tom Sumner, Thomas J. Scriba, Adam Penn-Nicholson, Mark Hatherill, Richard G. White

**Affiliations:** 1 TB Modelling Group, TB Centre, Centre for Mathematical Modelling of Infectious Diseases, Department of Infectious Disease Epidemiology, London School of Hygiene & Tropical Medicine, London, United Kingdom; 2 South African Tuberculosis Vaccine Initiative, Division of Immunology, Department of Pathology and Institute of Infectious Disease and Molecular Medicine, University of Cape Town, Cape Town, South Africa

**Keywords:** Tuberculosis, Epidemiology, Prognostic markers

## Abstract

Achieving the WHO End-Tuberculosis (TB) targets requires approaches to prevent progression to TB among individuals with *Mycobacterium tuberculosis (M.tb)* infection. Effective preventive therapy (PT) exists, but current tests have low specificity for identifying who, among those infected, is at risk of developing TB. Using mathematical models, we assessed the potential population-level impact on TB incidence of using a new more specific mRNA expression signature (COR) to target PT among HIV-uninfected adults in South Africa. We compared the results to the use of the existing interferon-γ release assay (IGRA). With annual screening coverage of 30% COR-targeted PT could reduce TB incidence in 2035 by 20% (95% CI 15–27). With the same coverage, IGRA-targeted PT could reduce TB incidence by 39% (31–48) but would require greater use of PT resulting in a higher number needed to treat per TB case averted (COR: 49 (29–77); IGRA: 84 (59–123)). The relative differences between COR and IGRA were not sensitive to screening coverage. COR-targeted PT could contribute to reducing total TB burden in high incidence countries like South Africa by allowing more efficient targeting of treatment. To maximise impact, COR-like tests may be best utilised in the highest burden regions, or sub-populations, within these countries.

## Introduction

Achieving the World Health Organisation (WHO) End-Tuberculosis (TB) strategy targets (reducing TB deaths by 95% and TB cases by 90%)^1^, and ultimately eliminating the disease, will not be possible without tools to prevent progression to TB among individuals with *Mycobacterium tuberculosis (M.tb)* infection^2^. Effective regimens for preventive therapy exist^3,4^ but treating the quarter of the global population estimated to be infected with *M.tb*^5^ is unlikely to be feasible, especially given the relatively low lifetime risk of developing disease and the rare, yet potential, adverse effects of prophylactic treatment.

Existing tests for *M.tb* infection, such as the tuberculin skin test (TST) and interferon-γ release assay (IGRA), have poor specificity for predicting who will progress to disease. New, more specific tests, which could more accurately identify those individuals at greatest risk of developing TB, and therefore allow better targeted preventive therapy, are needed. Several such tests are currently under development^6,7^ and the WHO have recently released a target product profile (TPP) outlining the desired characteristics of such a test^8^. The TPP identified those with increased likelihood of recent exposure (such as household contacts) or those with risk factors for developing TB (such as people living with HIV) as the primary target groups for such a test, especially in low-risk settings. However, in high incidence settings such tests may have value for screening the general population^8^.

An 11-gene version of a previously validated blood 16-gene mRNA expression signature^9^ is currently being evaluated in a proof-of-concept study in South Africa (Correlate of Risk (COR) Targeted Intervention Study (CORTIS-01; ClinicalTrials.gov NCT02735590)). This 11-gene version of the COR signature has been shown to have a specificity of 84% and sensitivity of 71% for predicting TB up to 1 year before the onset of disease in adolescents from South Africa^10,11^. The CORTIS-01 trial aims to further evaluate the diagnostic performance of the COR for identifying prevalent TB disease among adults who have not sought health care, and the prognostic performance of the COR for predicting incident TB disease in unselected populations. It also aims to test the efficacy of providing preventive therapy to COR-positive HIV-uninfected individuals in reducing TB incidence^11^.

Mathematical models, simplified representations of real world systems often as a system of differential equations, are increasingly used to study the spread and control of infectious diseases. In this paper we describe the results of a mathematical modelling study conducted to assess the potential population-level impact of COR targeted preventive therapy in a high TB incidence country using South Africa as an example. Despite declines in recent years, TB remains a major public health problem in South Africa. In 2015, there were an estimated 450,000 incident cases^12^ and TB was the leading cause of death due to an infectious disease^13^. While the majority of cases occur in HIV-infected individuals who, along with close contacts of TB cases, are recommended to receive preventive therapy^14–16^, there is still a significant burden in the HIV-uninfected adult population with an estimated TB incidence of 460/100,000 per year (see Appendix).

Using a dynamic transmission, in which the risk of infection depends on the simulated prevalence of disease, we estimate the long-term impact, accounting for both the direct benefit of preventing incident TB in those treated and the indirect effect of reducing future transmission, of COR targeted preventive therapy among the HIV-uninfected adult population. To assess the potential added benefit of more specific COR-like tests we compare the COR strategy to the use of IGRA to target preventive therapy.

## Methods

This paper reports the results of a mathematical modelling study related to the CORTIS-01 trial and does not report directly on any experiments using human samples. The CORTIS-01 trial protocol was approved by the University of Cape Town and all methods are conducted in accordance with relevant guidelines. All participants are over 18 years of age and written informed consent was obtained from all participants.

The model used only considers pulmonary forms of TB and does not include extra-pulmonary disease. Notification data reported by the WHO indicates that pulmonary diseases accounts for approximately 90% of all TB in South Africa. We assume that this proportion remains unchanged in the future.

### Preventive therapy strategies

We modelled 2 strategies for the future use of preventive therapy in South Africa and compared them to a base case of continuation of current practice in which preventive therapy is provided to HIV-infected individuals but is not routinely used in the HIV-uninfected population. In the first strategy we assume that HIV-uninfected adults are screened with COR while in the second strategy screening is conducted with IGRA. In both cases, those with a positive screening result are evaluated for TB to exclude active disease. Those found to have disease are offered treatment for active TB while those without disease are offered preventive therapy (isoniazid (INH) and rifapentine (RPT) for 3 months (3HP)).

### Modelling of a single round of screening

**Figure 1:**
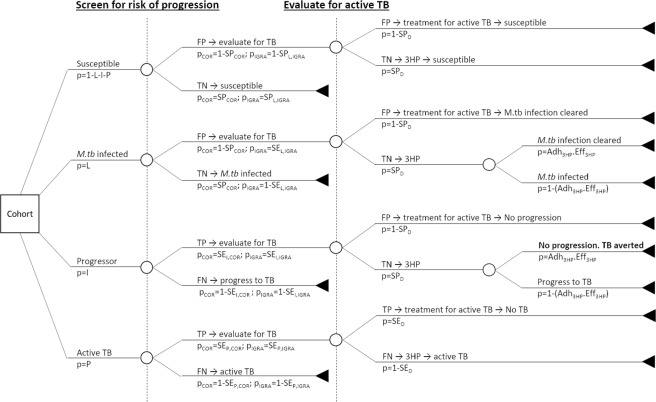
Decision tree for the preventive therapy strategy. Individuals who screen positive on COR/IGRA are evaluated for active TB. Individuals found to have active TB are treated for disease, those found not to have active TB are provided 3HP. 3HP prevents progression to disease with a probability given by the adherence (Adh_3HP_) and efficacy (Eff_3HP_). SP_COR_ = specificity of COR; SE_I,COR_ = sensitivity of COR for progression to TB; SE_P,COR_ = sensitivity of COR for prevalent active TB; SP_L,IGRA_ = specificity of IGRA for *M.tb* infection; SE_L,IGRA_ = sensitivity of IGRA for *M.tb* infection; SE_I,IGRA_ = sensitivity of IGRA for progression to TB; SE_P,IGRA_ = sensitivity of IGRA for prevalent active TB; SP_D_ = specificity of evaluation for active TB; SE_D_ = sensitivity of evaluation for active TB; FP = false positive; TP = true positive; FN = false negative; TN = true negative. Full details of parameter values are given in Table 1.

For each strategy, we first calculated the outcomes from a single round of screening in a cohort. The population was divided into those with no history of exposure (susceptibles, *S*) those with *M.tb*-infection but not at risk of developing TB in the following year (*L*), those who will become incident TB cases in the next year (progressors, *I*) and those with prevalent active TB (*P*). The strategy is illustrated in Fig. 1. Details of parameters are given in Table 1.

**Table 1:** Parameters used to estimate outputs from a single round of screening.

Parameter	Description	Value (mean and 95% CI)	Source
*SE*_*P,COR*_	Sensitivity of COR for prevalent TB	0.91 (0.78–0.97)	Darboe *et al*.^10^
*SE*_*P,IGRA*_	Sensitivity of IGRA for prevalent TB	0.84 (0.78–0.91)	Metcalfe *et al*.^30^
*SE*_*I,COR*_	Sensitivity of COR for progression	0.71 (0.57–0.82)	Zak *et al*.^9^, Penn-Nicholson *et al*.^31^, Fiore-Gartland *et al*.^11^
*SE*_*I,IGRA*_	Sensitivity of IGRA for progression	0.72 (0.58–0.82)	Rangaka *et al*.^32^
*SP*_*COR*_	Specificity of COR for progression*	0.84 (0.79–0.88)	Penn-Nicholson *et al*.^31^, Fiore-Gartland *et al*.^11^
*SE*_*L,IGRA*_	Sensitivity of IGRA for *M.tb* infection	0.78 (0.73–0.82)	Pai *et al*.^33^
*SP*_*L,IGRA*_	Specificity of IGRA for *M.tb* infection	0.96 (0.94–0.98)	Pai *et al*.^33^
*SE*_*D*_	Sensitivity of diagnosis (Xpert)	0.89 (0.81–0.94)	Steingart *et al*.^34^
*SP*_*D*_	Specificity of diagnosis (Xpert)	0.99 (0.96–1)	Steingart *et al*.^34^
*Adh*_*3HP*_	Proportion of those starting 3HP who complete (used in NNT calculation)	0.82 (0.61–0.95)	Sterling *et al*.^4^, Sandgren *et al*.^35^
*Eff*_*3HP*_	Efficacy of 3HP for preventing progression to TB in those who complete (used in NNT calculation)	0.6 (0.48–0.7)	Smieja *et al*.^3^. Assumes that 3HP is non-inferior to isoniazid^4^.
*L*	Proportion of population *M.tb* infected	0.5 (0.2–0.8)	Assumption
*I*	Proportion of population who will progress to TB (incidence)	0.0046 (0.0028–0.0064)	Calculated for HIV-uninfected adults from WHO incidence estimates (see Appendix)
*P*	Proportion of population with prevalent TB	0.0076 (0.0046–0.0106)	Calculated for HIV-uninfected adults from WHO incidence estimates (see Appendix)

Confidence intervals based on ACS/SHIP data were calculated using Wilson score interval. All parameters were assumed to follow beta distributions. Shape and scale parameters were estimated from median and 95% CI using the rriskdistributions package in R^36^. The susceptible population, *S* = 1 − *L* − *I* − *P*. *The specificity of COR is assumed to be independent of true infection status.

We simulated 10,000 cohorts with parameters sampled independently from the distributions specified in Table 1. From these outputs we calculated the positive predictive value (PPV) for prevalent and incident TB, the number needed to screen and test to diagnose one prevalent case, and the number needed to treat with 3HP to avert one TB case (assuming adherence to and efficacy of 3HP as indicated in Table 1).

### Dynamic transmission modelling of multiple rounds of screening

**Figure 2:**
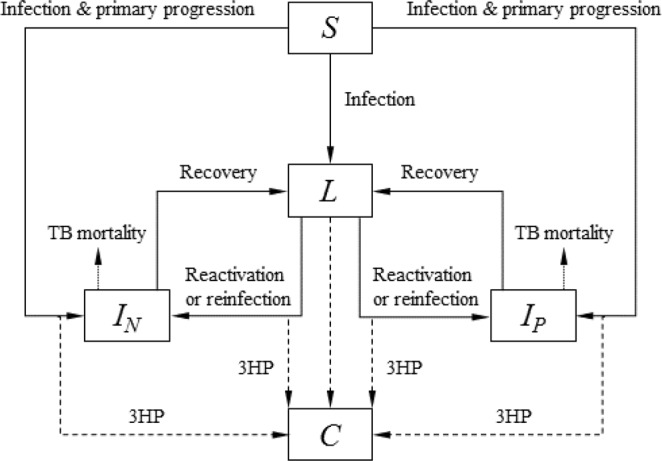
Model structure. Core structure of the transmission model. *S* = susceptible, *L* = latently infected, *I*_*N*_ = smear negative TB, *I*_*P*_ = smear positive TB, *C* = post 3HP. Susceptible individuals are infected at a rate that depends on the prevalence of active disease. Following infection, some proportion progress directly to active disease (primary progression), the remainder entering the *M.tb* infected state. Infected individuals may remain infected, progress to disease (reactivation) or be re-infected, with some reduced risk of developing primary disease due to their prior infection. Individuals with active disease can die of TB, self-cure, or be diagnosed and treated for TB (recovery). Dashed arrows indicate the reduction in progression to TB (or treatment of *M.tb* infection) as a result of preventive therapy (3HP). For clarity the age structure, background mortality and HIV structure are not shown in the figure.

We then used an age structured dynamic transmission model to simulate the long-term population-level impact of each of the strategies. This allows us to capture the indirect effect of preventing incident cases on future *M.tb* transmission. We also estimated the amount of testing, TB treatment and 3HP required in each strategy. The model, which is similar in structure to a number of published TB models^17,18^, is illustrated schematically in Fig. 2. Full details of the model can be found in the Appendix. As drug resistance is not the focus of the study, for simplicity we do not include it in the model. Although the preventive therapy strategies are targeted at HIV-uninfected adults, the transmission model includes the HIV-infected population and children to allow us to assess the impact on the overall TB burden. Age-specific HIV incidence and ART coverage are time varying external inputs to the model. The HIV-infected population is stratified by CD4 count and time on ART. The risk of developing TB and dying from the disease depend on CD4 count and ART status. The baseline model was calibrated to the WHO-estimated TB incidence and mortality (rate/100,000), TB notifications, the proportion of incident TB in HIV-infected individuals and the proportion of TB in children (<15 years of age) in South Africa using a sampling-importance-resampling approach. Further details of the model and the calibration methods are given in the Appendix.

The screening and preventive therapy strategies are modelled as for the single round of screening described above (see Fig. 1 and Table 1). In our main analysis we assumed that 30% of the HIV uninfected adult population were screened per year. We also modelled lower coverages of 5, 10 and 20% to explore how the results depend on coverage. In all cases, screening is assumed to be random.

Those identified as prevalent active TB cases (both true positive and false positive) start treatment with probability *κ* (to account for pre-treatment loss to follow up). Among true positive prevalent cases who start treatment, a proportion *τ* are successfully treated and return to the “*M.tb*-infected” state in the model. Those who are not successfully treated remain as prevalent active TB cases.

Those identified by the tests as potential progressors (both true positive and false positive) start preventive therapy with probability *κ*. Among true positive progressors, 3HP is effective with a probability given by the product of the adherence and efficacy. These individuals move to the “post-3HP” state (see below). Those in whom 3HP is not effective become incident TB cases.

*M.tb-*infected individuals, who will not progress to TB in the next year, and who successfully complete treatment or 3HP move to the “post-3HP” state (see below). Neither treatment for TB or 3HP is assumed to have an effect in “susceptible” individuals (i.e. those with no exposure to *M.tb*).

In our primary analysis, those in the post-3HP state are assumed to be “cured” of their infection, and therefore are not at risk of developing reactivation TB, but can be re-infected and either develop primary TB or return to the *M.tb-*infected state (with the same assumed level of protection against primary disease following reinfection as those in the *M.tb-*infected state). Individuals may receive more than one course of 3HP during the timeframe considered. In addition, we assume that treatment for *M.tb* infection does not change the response to IGRA or COR^19^. To explore the importance of key assumptions around preventive therapy we carried out the following scenario-based sensitivity analyses: assuming that individuals are still *M.tb-*infected following 3HP; only allowing for a single course of 3HP in any individual.

The dependence of the model predictions on the input parameters were assessed by calculating partial rank correlation coefficients (PRCCs) for each input parameter. We also visually assessed the relationship between intermediate model outputs (TB incidence, prevalence of infection, annual risk of infection, proportion of disease due to recent transmission, proportion of incident TB in HIV-infected and proportion of TB in children) and the model predictions using scatter plots.

## Results

### Modelling of a single round of screening

Table 2 shows the results of a single round of screening. Using IGRA, 75% of prevalent TB cases are correctly diagnosed, while in the COR scenario this increases to 81%. Both strategies result in a similar proportion of progressors (approximately 70%) correctly being given 3HP. The IGRA strategy involves more testing for TB, treatment of active TB and 3HP compared to the COR strategy. Approximately 40% of the population would be tested for active TB and offered 3HP in the IGRA scenario compared to approximately 16% based on COR. As a result, the positive predictive value (PPV) for both provision of 3HP and treatment of active TB is higher for COR: approximately 2% of those offered 3HP based on the COR would have progressed to TB in the next year compared to less than 1% when using IGRA. Similarly, 79% of those treated for active TB based on COR are true prevalent TB cases compared to 59% for IGRA (see Table 2).

**Table 2:** Model results.

	COR	IGRA
**SINGLE ROUND OF SCREENING**		
**Tested for TB after screening (target group** = **prevalent cases**, ***P*)**		
% of total population	16.8 (12.9–21.5)	42.0 (20.2–64.5)
% of target group	91.4 (81.2–97.3)	84.1 (77.7–89.2)
Positive predictive value (PPV) for prevalent TB	4.1 (2.4–6.3)	1.5 (0.8–3.4)
**Treated for TB (target group** = **prevalent cases**, ***P*)**		
% of total population	0.8 (0.5–1.3)	1.0 (0.5–2.4)
% of target group	80.9 (70.4–88.7)	74.5 (66.6–81.4)
Positive predictive value (PPV) for prevalent TB	78.5 (46.0–97.8)	58.7 (21.8–95.0)
Number screened to diagnose one prevalent TB case	163.9 (115.2–275.7)	177.4 (124.3–300.9)
Number tested to diagnose one prevalent TB case	27.7 (17.8–48.0)	74.1 (32.6–145.6)
**Given 3HP (target group** = **progressors**, ***I*)**		
% of total population	16.0 (12.0–20.6)	40.7 (18.8–63.1)
% of target group	69.8 (56.5–81.0)	70.9 (58.5–81.6)
Positive predictive value (PPV) for progression to TB	2.0 (1.1–3.1)	0.8 (0.4–1.8)
Number given 3HP to avert one incident TB case	109.7 (63.3–209.7)	272.4 (114.5–589.0)
**TRANSMISSION MODEL**		
**% Reduction in TB incidence**		
2020	5.1 (3.5–7.2)	5.6 (3.9–7.7)
2035	20.4 (15.2–26.9)	38.8 (31.2–48.0)
**Cumulative cases averted (thousands)**		
During 2020	13 (8–20)	14 (9–22)
2020 to 2035	490 (318–767)	839 (541–1296)
**“Resources” used (yearly average 2020 to 2035)**		
Tested for TB (millions)	1.8 (1.3–2.4)	5.5 (4.1–6.4)
Treated for TB (thousands)	12.6 (8.8–18.2)	19.6 (13.8–26.6)
Given 3HP (millions)	1.5 (1.1–1.9)	4.6 (3.4–5.3)

As expected, the PPV for 3HP is strongly dependent on the sensitivity and specificity of the tests (COR or IGRA) and the incidence of TB disease (see Appendix Fig. A.2). For IGRA, the PPV is also strongly determined by the prevalence of infection in the population: at a lower prevalence of infection the PPV of IGRA would increase. However, for the sensitivity and specificity assumed here, the prevalence of infection would have to be below 12% for the PPV of IGRA to exceed that of the COR (see Appendix Fig. A.3). This finding may have relevance for the use of COR like assays in low incidence settings. Of those incorrectly given 3HP (i.e. they are not true progressors) approximately 50% are susceptible and 50% *M.tb-*infected based on the COR. However, in the case of IGRA the majority (approximately 95%) are *M.tb-*infected and potentially could accrue some long-term benefit from preventive therapy (see Appendix Fig. A.1).

### Dynamic transmission modelling of multiple rounds of screening

**Figure 3:**
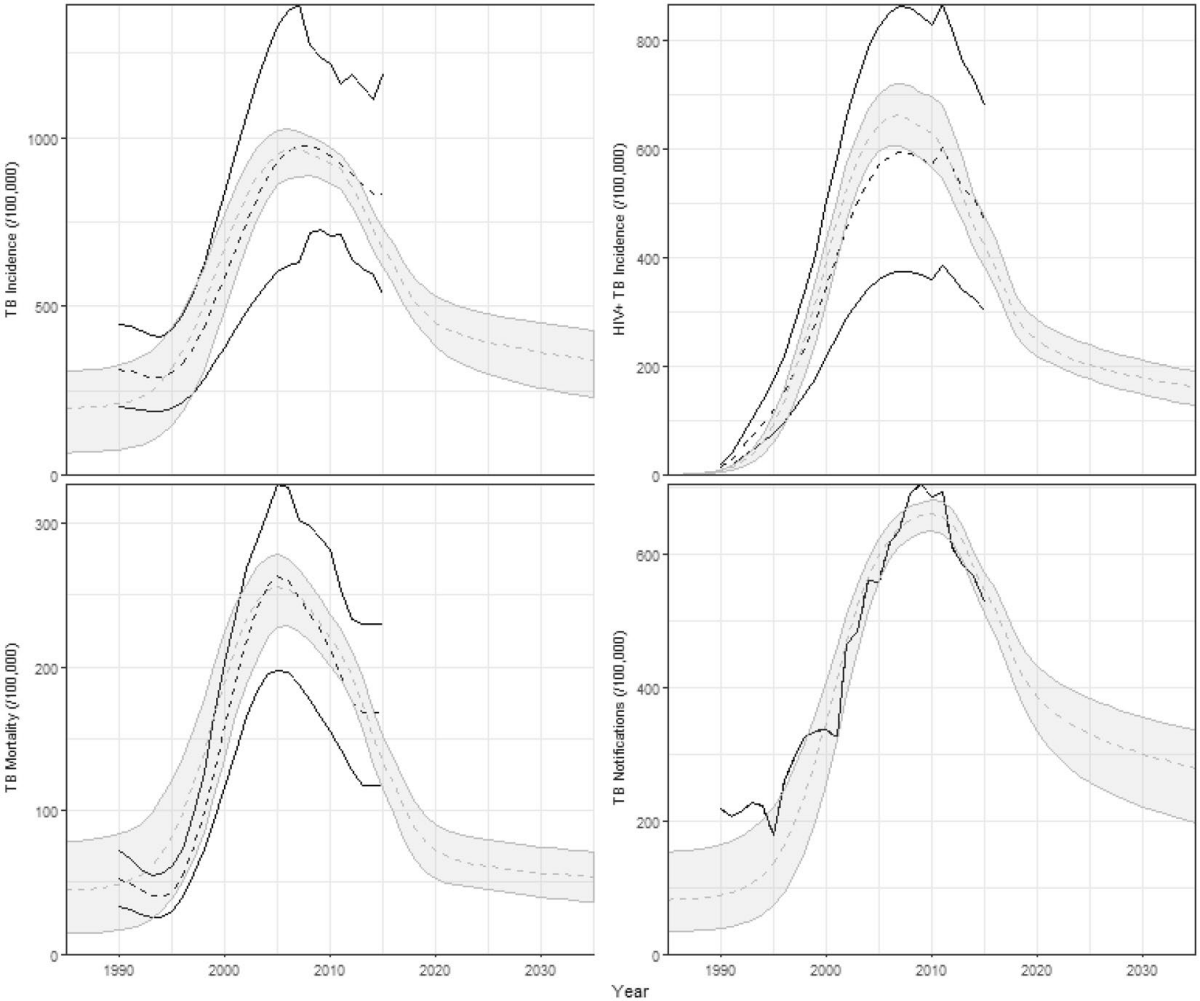
Baseline fit of the model. Black lines show median (dashed) and range (solid lines) of WHO estimates of TB incidence and mortality and reported notifications. Shaded areas show 95% confidence interval of the model outputs.

Figure 3 shows the baseline fit of the transmission model. The model fits to historical estimates of TB incidence and mortality, reported TB notifications and the disaggregation of TB incidence by HIV status. The modelled proportion of TB in children <15 years of age is 9·9–15·4%, consistent with the estimates reported by WHO (13·2% (6·4–25·3%), see Appendix Fig. A.7). The model predicts that with continuation of current practice, and ongoing scale-up of ART, TB incidence will decline to approximately 372/100,000 (248–432) by 2035.

**Figure 4:**
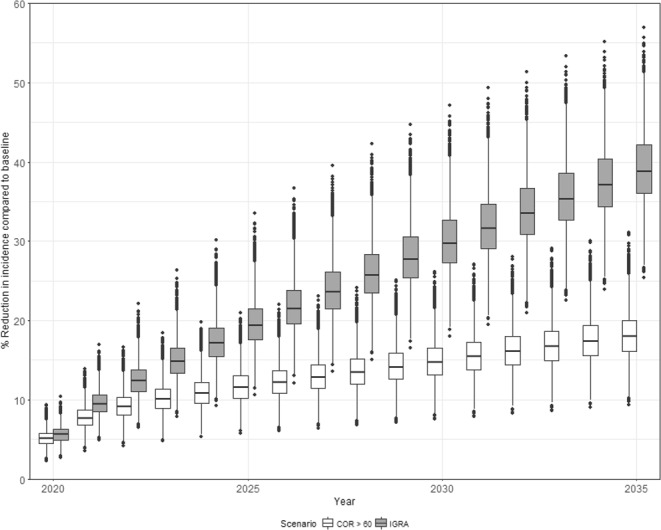
Impact of targeted 3HP. Percentage reduction in TB incidence rate compared to baseline (y-axis) as a function of year (x-axis). Shading indicates the strategy. Boxes show the median and interquartile range (IQR). Whiskers show the largest values (or 1.5 IQR). Dots indicates outliers.

Figure 4 shows the percentage reduction in TB incidence compared to the baseline for each strategy over time assuming 30% coverage. This represents the additional reduction in incidence due to the strategy on top of the baseline decline shown in Fig. 3. The impact of a single round of screening (in 2020) is similar across both strategies at approximately 5% (see Table 2). The impact increases over time, reaching 20% (15–27%) by 2035 using COR. The long-term impact of IGRA-targeted 3HP is larger, reaching 39% (31–48%) by 2035. In both cases, the majority of the impact results from preventing incident cases via preventive therapy and less from identifying and treating prevalent TB (see Appendix section A.3.2 for a discussion of this finding).

**Figure 5:**
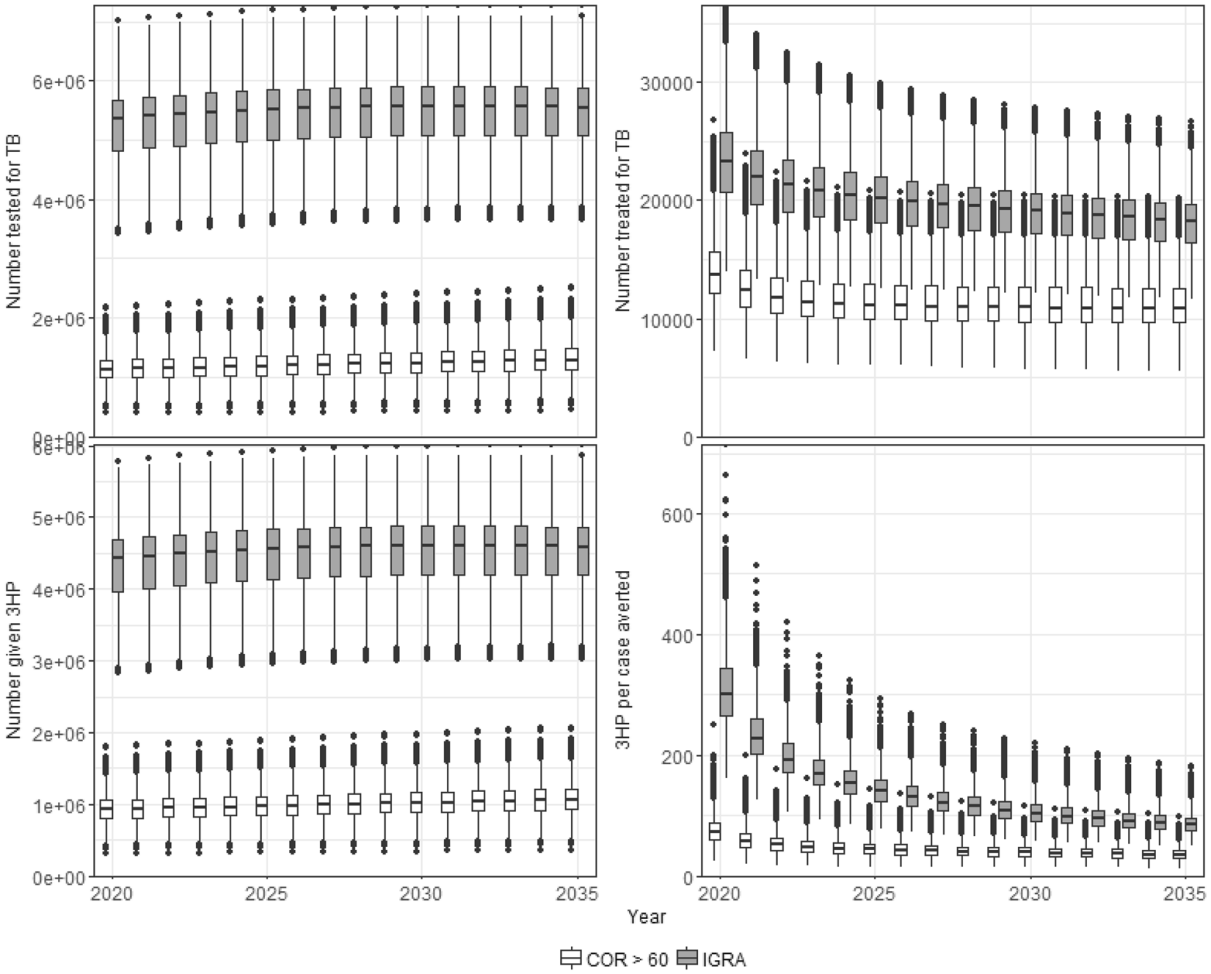
Resources used and number needed to treat. (**A**) Number tested for TB (top left); (**B**) number diagnosed and started on TB treatment (top right); (**C**) number given 3HP (bottom left) and (**D**) number needed to treat with 3HP per case averted (cumulative to year indicated). Shading indicates strategy. Boxes show the median and interquartile range (IQR). Whiskers show the largest values (or 1.5 IQR). Dots indicates outliers.

However, IGRA-based strategies involve far greater volumes of testing and treatment for TB and 3HP as illustrated in Fig. 5A–C. Approximately 5.5 million people must be tested for TB and 4.6 million people must be treated with 3HP per year using IGRA, compared with approximately 1.8 million tested and 1.5 million given 3HP if using COR.

Figure 5D shows the number needed to treat (NNT) with 3HP to avert one case of TB. This is calculated as the cumulative number given 3HP, divided by the cumulative number of cases averted up to a given time. The NNT is much lower for COR-based strategies compared to IGRA after a single round (107 (64–178) vs 300 (211–442)). These are consistent with the values calculated in the simple model of a single round of screening (see Table 2). By 2035 the cumulative NNT for IGRA falls to 84 (59–123) but is still higher than that of COR-based strategies (NNT = 49 (29–77)).

For comparison, we also used the transmission model to estimate the potential impact of a test with the optimal characteristics defined in the WHO TPP^8^. Assuming a sensitivity and specificity of 90% we found a similar impact to that achieved with COR (6.1% (4.2–8.3%) after a single round of screening; 19.3% (14.4–25.4%) by 2035). However, due to the more specific targeting of preventive therapy to those at short term risk of progression the cumulative NNT for the optimal TPP test was lower at 30 (19–46).

As expected, the reduction in incidence and the number of cases averted are lower at lower screening coverage for both COR and IGRA. In contrast the NNT increases slightly as coverage increases; the larger impact achieved with higher coverage means that the prevalence of TB is lower and hence the NNT is higher. Importantly, the relative difference between the COR and IGRA strategies is largely independent of screening coverage. In 2020 the NNT for IGRA is 2.8 times that for COR for all coverages considered. The cumulative NNT by 2035 using IGRA is 1.5 times that of COR at 5% coverage rising to 1.7 times that of COR at 30% coverage. Full details of these results can be found in Appendix Table A.9.

The probabilistic sensitivity analysis (Appendix Fig. A.8) suggests that, for the ranges of parameters considered here, the impact is most strongly determined by the adherence to, and efficacy of, 3HP. The impact is also positively correlated with the annual risk of infection suggesting that the impact of COR may be increased in settings with higher levels of ongoing transmission (Fig. A.9). For further details, see the Appendix.

The results of the scenario analysis are shown in Appendix Figs A.11–A.15. The predicted impact of all strategies is sensitive to both the assumed effect of 3HP on infection status (cure vs no cure) and the number of courses of 3HP an individual can receive (multiple vs single). Allowing only a single course of 3HP per individual reduces the impact of all strategies, however the long-term impact of using IGRA still exceeds that of using COR. Under the ‘no-cure’ assumption, the impact of all strategies is approximately the same. When these assumptions are combined (no cure, single course of 3HP) the predicted impact of IGRA-targeted 3HP is lower than that of a COR-based strategy (Fig. A.11). Only allowing a single round of 3HP also results in a drop in the number of doses of 3HP required per year; this drop is greatest in the IGRA-based strategy, resulting in comparable levels of 3HP in all strategies by 2035 (see Fig. A.14). However, given the corresponding reduction in predicted impact, the NNT with 3HP to avert one TB case remains higher for IGRA (compared to COR) for all combinations of these assumptions (see Fig. A.15).

We also considered the impact of the strategies in the absence of 3HP, i.e. what is the impact of COR or IGRA as a test for identifying prevalent TB. These results are shown in the Appendix (Table A.10). For all strategies, the impact of identifying prevalent cases is predicted to be relatively small (~3.5%) and a minority of the overall impact when PT is included. However, it should be noted that this finding is probably dependent on our assumptions that all prevalent cases have the same probability of being diagnosed and that infectiousness is assumed to be constant throughout the entire duration of disease. If the prevalent cases identified by COR (or IGRA) are those who are at the early stages of subclinical or asymptomatic disease and/or those who would not be identified via current screening approaches, the impact could be greater. In contrast, if the cases identified by COR would then not be correctly diagnosed using the current South African algorithm (Xpert) the impact could be reduced. While previous modelling has shown that these factors will influence the potential impact of a new diagnostic^20^ we currently do not have sufficient data to inform the model and have chosen to use the simplest possible approach here

## Discussion

These results suggest that COR-targeted preventive therapy in HIV-uninfected adults could make an important contribution to reducing the TB burden in high incidence countries such as South Africa, with estimated reductions in incidence of 20% (15–27%) over 15 years. While the model suggests that IGRA-targeted 3HP could result in greater reductions in incidence (39% (31–48%)) over the same period, the number of people who would be treated with 3HP per year is significantly higher (approximately 4.6 million for IGRA vs 1.5 million for COR). When calculated over the time-period considered, the NNT to avert one TB case using IGRA is 1.7 times that for COR (84 (59–123) vs 49 (29–77)).

The recently released TPP for tests to predict progression of TB indicated that those with increased likelihood of recent exposure or risk factors for progression (such as household contacts or HIV-infected people) should be the primary target groups for testing. Our results suggest that in high incidence settings such as South Africa, the use of such tests in the general population may have potential. While the estimated PPV of COR targeted 3HP is relatively low at around 2%, it is approximately double that for IGRA. To put these values in context, it is useful to compare to the PPV among high-risk sub-populations recommended to receive preventive therapy in South Africa. For example, WHO and national guidelines suggest that all HIV-infected individuals, irrespective of ART status, should receive preventive therapy^14,15^. In 2015 there were an estimated 258,000 incident TB cases among the estimated 7 million HIV-infected individuals in South Africa: an incidence, and hence PPV of preventive therapy, of approximately 3·7%^21,22^.

However, the resources required to screen 30% of the population per year and provide preventive therapy to approximately 1.5 million people are likely to be prohibitive. As such, the use of tests such as the COR to screen the general population may be best focussed on the highest incidence districts to maximise impact. Such tests may also provide additional benefit among those already recommended to receive preventive therapy, including HIV-infected individuals and household contacts of TB cases, by identifying those at most immediate risk of progression to disease.

Sensitivity analysis suggests that the long-term impact of 3HP, especially in the IGRA strategy, is dependent on the assumption that 3HP “cures” *M.tb* infection. Our previous analysis of preventive therapy trials in HIV-infected populations has suggested a potential lack of “cure” in this group^23,24^ but it is likely that this is not the case in HIV-uninfected people.

As with any model, we have made several simplifying assumptions which may affect the conclusions. Uptake and completion of preventive therapy among HIV-infected individuals has been challenging in South Africa^25^, and similar challenges are likely to be experienced in targeted use of 3HP among HIV-uninfected populations. We assumed uptake of 3HP following a positive COR or IGRA would be the same as initiation of treatment among those diagnosed with active TB which may overestimate the potential impact. Similarly, our assumed values of adherence may overestimate what may be observed in programmatic use. We have not included uncertainty in future projections of HIV incidence in South Africa. The impact of a strategy targeted at HIV-uninfected adults will be affected by the future HIV dynamics. We did not include drug resistance in our model as it was not the primary focus of this research. However, approximately 3.5% of TB cases in South Africa are estimated to have multi-drug resistant or rifampicin-resistant forms of TB^21^. This would reduce the overall impact predicted here, assuming 3HP is ineffective in preventing progression to drug resistant TB, but should not significantly affect the relative difference between COR and IGRA based strategies. There are also concerns that population-level preventive therapy may lead to increased drug resistance. However, previous studies have found no significant association between isoniazid preventive therapy (IPT) and development of drug resistance^26^ and modelling has suggested that the timescales over which community wide IPT could drive increases in resistance are longer than those considered here^27^.

Based on available data^28,29^, we have assumed that 3HP does not affect IGRA or COR status and as such those who have received effective 3HP will be identified as potential recipients if subsequently screened. If this is not the case, this is likely to result in an overestimate of the amount of 3HP required in the IGRA strategies and hence may overestimate the long-term NNT for this strategy. We also assumed that COR false positives are equally distributed between *M.tb-*infected and -uninfected individuals which will similarly inflate the NNT estimates for COR. Results from the CORTIS-01 study will provide estimates for these variables and reveal the relevance of these assumptions.

## Conclusion

Targeting 3HP based on a COR-like test could have an important impact on TB incidence and, in HIV-uninfected individuals, is likely to be more efficient than using existing tests for *M.tb* infection such as IGRA. In low-income countries, the need to screen and treat large populations may be prohibitive and such tests may be best utilised in the highest burden regions within these countries.

## Supplementary information

Appendix
