# Supplementary material for: Potential population level impact on tuberculosis incidence of using an mRNA expression signature correlate-of-risk test to target tuberculosis preventive therapy
Source: Sci Rep. 2019 Jul 31;9:11126. doi: 10.1038/s41598-019-47645-z (PMC6668474; doi:10.1038/s41598-019-47645-z)
Supplement: Supplementary file 1 — Appendix [file 41598_2019_47645_MOESM1_ESM.docx]

**Potential population level impact of correlate-of-risk targeted preventive therapy on Tuberculosis**

Tom Sumner^1^, Thomas J. Scriba^2^, Adam Penn-Nicholson^2^, Mark Hatherill^2^, Richard G. White^1^

**Affiliations:**

^1^TB Modelling Group, TB Centre, Centre for the Mathematical Modelling of Infectious Disease, Department of Infectious Disease Epidemiology, London School of Hygiene & Tropical Medicine, United Kingdom

^2^South African Tuberculosis Vaccine Initiative and Institute of Infectious Disease and Molecular Medicine and Division of Immunology, Department of Pathology, University of Cape Town, Cape Town, South Africa.

**Appendix**

**A.1 Additional details and results from the single round of screening model**

A.1.1 Estimating adult HIV uninfected TB incidence and prevalence 2

A.1.2 Additional results from simple model 3

**A.2 Details of the transmission model**

A.2.1 TB Model 6

A.2.2 HIV Model 9

A.2.3 Demographic model 14

A.2.4 Model equations 15

A.2.5 Model calibration 17

**A.3** **Additional transmission model outputs**

A.3.1 Baseline fit 18

A.3.2 Varying screening coverage 22

A.3.3 Sensitivity analysis 23

A.3.4 Scenario analysis 27

**A.1 Additional details and results from the single round of screening model**

**A.1.1 Estimating adult HIV uninfected TB incidence and prevalence**

To generate results from the simple model of a single round of screening we had to estimate the incidence and prevalence of TB in the target population (adult (>15 years of age), HIV uninfected) in South Africa. The assumptions used to make this estimate are described below.

| **Parameter** | **Value** | **Source** |
| --- | --- | --- |
| Total population of South Africa | 56 million | UN population division |
| Percentage of population >15 yrs | 29% | World Bank |
| HIV infected adults | 6.8 million | UNAIDS |
| Incident TB cases | 438,000 | WHO TB database |
| HIV infected incident TB cases | 258,000 | WHO TB database |
| <15 incident TB cases | 180,000 | WHO TB database |
| Percentage of <15 TB cases HIV infected | 50% | Venturini et al, BMC ID, 2014 |
| Average duration of non-notified TB case | 2.5 years | WHO methods for estimation |
| Average duration of notified TB case | 1.1 years | WHO methods for estimation |
| Percentage of cases notified | 60% | WHO TB database |

**Table A.1.** Data used in the estimation of adult HIV-uninfected TB incidence and prevalence in South Africa

Given the total population, the percentage over 15 yrs of age and the number of HIV infected adults we can estimate the HIV uninfected adult population as:

((1-0.29) x 56,000,000) – 6,800,000 = 32,960,000

Similarly, we can estimate the number of incident TB cases in HIV uninfected adults as

(438,000-258,000) – (1-0.5) x 58,000 = 151,000

Therefore, the rate of TB incidence in HIV uninfected adults is 460/100,000

Using the approximate relationship that Prevalence = Incidence x Duration of disease, the prevalence of TB in HIV uninfected adults is given by:

460 x ((0.6 x 1.1 + (1-0.6) x 2.5) = 760/100,000

To estimate approximate ranges for these values we used the uncertainty in WHO estimated incidence of TB in South Africa (+/- 40%) and applied this variability to the estimates above.

**A.1.2 Additional results from simple model**

Figure A.1 shows how testing for TB, treatment for TB and 3HP are distributed across the four population groups (susceptible, latently infected, progressors and prevalent active TB cases) for each of the strategies (COR, IGRA). For COR based strategies, a greater proportion of both treatment for TB disease (centre panel) and 3HP (right panel) is given to the correct population groups (prevalent cases and progressors respectively). However, a greater proportion of both TB treatment and 3HP is also given to susceptible individuals, i.e. those with no exposure to *M.tb* compared to the IGRA strategy.


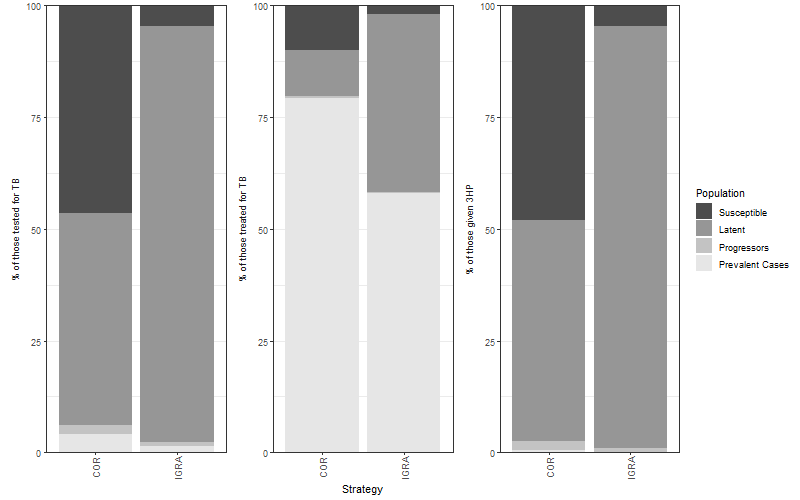


**Figure A.1** Distribution of a) testing for TB, b) TB treatment and c) 3HP across the 4 population subgroups (indicated by shaded areas) for each of the strategies (shown on the x-axis).


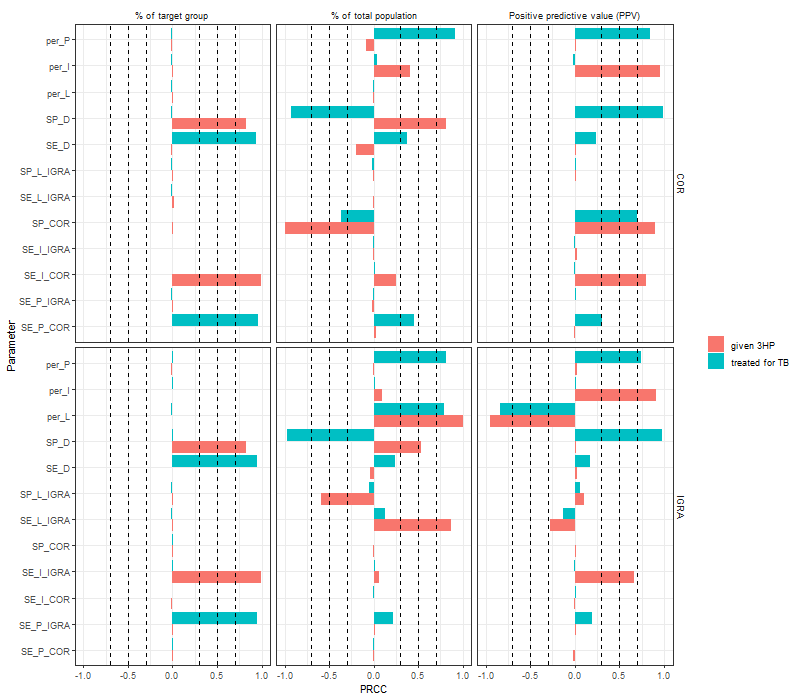


**Figure A.2.** Partial rank correlation coefficients (PRCCs) for the simple model of a single round of screening. Parameters in the model are indicated on the y-axis, PRCC values on the x-axis. Blue bars show results for the proportion treated for TB, red bars for the proportion given 3HP. Positive values indicate a positive correlation, negative values a negative correlation. Dashed lines indicate commonly used thresholds for weak (±0.3), moderate (±0.5) and strong (±0.7) correlations. The 2 screening strategies are shown by row (top = COR; bottom = IGRA). Columns show the different outputs of interest (left=proportion of target group; middle=proportion of total population; right=positive predictive value (PPV)).


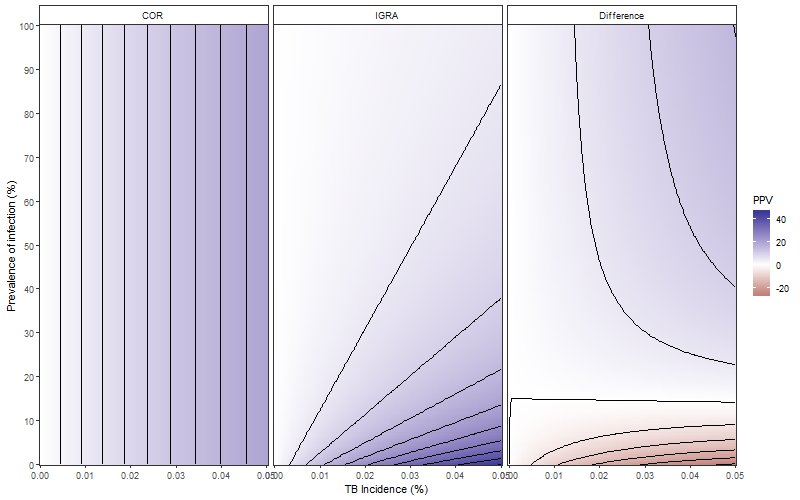


**Figure A.3.** Contour plot showing the PPV of 3HP based on a) COR, b) IGRA and c) the difference in PPV between COR and IGRA, as a function of the prevalence of latent TB infection (y-axis) and TB incidence (x-axis)

Figure A.3 shows the PPV as a function of TB incidence and prevalence of latent infection. The left panel illustrates that for COR the PPV is independent of infection prevalence and depends only on TB incidence, increasing as TB incidence increases. This is due to the assumption that false positive COR positives are equally likely to be susceptible or latently infected ([1](#_ENREF_1)). The central panel shows the PPV based on IGRA which depends on both prevalence of infection and incidence of TB disease. The right panel shows the difference between the PPV of the two tests. Negative (red) values indicate where IGRA has a higher PPV than COR while blue areas indicate where COR is superior to IGRA. From the right panel we can see that, given the assumed sensitivity and specificity of COR and IGRA, if the prevalence of infection is greater than approximately 12% then COR has an equal or higher PPV than IGRA.

**A.2 Details of the transmission model**

The model used in this exercise is similar in structure to a number of published TB models ([2](#_ENREF_2), [3](#_ENREF_3)). The model is age structured (by 5-year age groups) and includes HIV and ART.

**A.2.1 TB model**

The population is divided into 3 main “TB” states: susceptible, latently infected, and active disease (stratified into smear positive and smear negative states). Susceptible individuals are infected at a rate that depends on contact between individuals and the prevalence of active disease in the population. Following infection, some proportion progress directly to active disease, with the remainder entering the latent state. Latently infected individuals may remain infected, progress to disease (reactivation), be re-infected or incorrectly treated for TB. Individuals with active disease can self-cure, die or be diagnosed and treated for TB. Table A.2 lists the TB states.

| **Symbol** | **Description** |
| --- | --- |
| *S* | Susceptible |
| *L* | Latently infected with drug susceptible strain, no previous treatment history |
| *N* | Smear negative, drug susceptible TB, no previous treatment history |
| *I* | Smear positive, drug susceptible TB, no previous treatment history |
| *C* | Latently infected, incorrectly treated for TB (no previous treatment) |

**Table A.2.** TB states in the model

The following table (A.3) details the natural history parameters used in the model.

| **Parameter** | **Description** | **Values** | **Source** |
| --- | --- | --- | --- |
| *β* | Effective contact rate | 10-20 | - |
| *a* | Proportion developing primary TB | 0.08-0.15 | ([4-6](#_ENREF_4)) |
| *v* | Reactivation rate | 0.001-0.0025 | ([4](#_ENREF_4), [6](#_ENREF_6), [7](#_ENREF_7)) |
| *p* | Protection against TB due to prior infection | 0.37-0.9 | ([4](#_ENREF_4)) |
| *σ* | Proportion developing smear positive TB, among HIV uninfected | 0.4-0.5 | ([8](#_ENREF_8)) |
| *σH* | Proportion developing smear positive TB, among HIV infected | 0.2-0.4 | ([9](#_ENREF_9), [10](#_ENREF_10)) |
| *ω* | Relative infectiousness of smear negative TB | 0.16-0.32 | ([11](#_ENREF_11), [12](#_ENREF_12)) |
| *ϑ* | Smear conversion rate among HIV uninfected | 0.007-0.03 | ([7](#_ENREF_7), [13](#_ENREF_13)) |
| *ϑH* | Smear conversion rate among HIV infected | 0.007-0.03 | Assumed same as HIV uninfected |
| *c_I_* | Case fatality in untreated smear positive TB, among HIV uninfected | 0.55-0.75 | ([14](#_ENREF_14)) |
| *c_N_* | Case fatality in untreated smear negative TB, among HIV uninfected | 0.1-0.3 | ([14](#_ENREF_14)) |
| *d* | Average duration of untreated TB, among HIV uninfected | 1.5-2.5 | ([14](#_ENREF_14), [15](#_ENREF_15)) |
| *cH_I_* | Case fatality in untreated smear positive TB, among HIV uninfected | 0.7-0.99 | ([14](#_ENREF_14)) |
| *cH_N_* | Case fatality in untreated smear negative TB, among HIV uninfected | 0.63-0.9 | ([14](#_ENREF_14)) |
| *dH* | Average duration of untreated TB, among HIV infected | 0.25-1 | ([14](#_ENREF_14), [15](#_ENREF_15)) |
| *μ_I_* | Mortality rate in smear positive TB, among HIV uninfected | *c_I_*/*d* | - |
| *μ_N_* | Mortality rate in smear negative TB, among HIV uninfected | *c_N_*/*d* | - |
| *r_I_* | Self-cure rate in HIV neg, smear pos | (1/*d*)-*μ_I_* | - |
| *r_N_* | Self-cure rate in HIV neg, smear neg | (1/*d*)-*μ_N_* | - |
| *μH_I_* | Mortality rate smear pos, HIV pos | *cH_I_*/*dH* | - |
| *μH_N_* | Mortality rate smear neg, HIV pos | *cH_N_*/*dH* | - |
| *rH_I_* | Self-cure rate in HIV pos, smear pos | (1/*dH*)-*μH_I_* | - |
| *rH_N_* | Self-cure rate in HIV pos, smear neg | (1/*dH*)-*μH_N_* | - |
| *H* | HIV Incidence | External input | UNAIDS |
| *m* | Migration | External input | DemProj |
| *μ* | Background mortality | External input | UN population projections 2015 update |

**Table A.3.** TB model parameters. In the absence of treatment, the CFR is given by μ/(μ+r) and the duration of disease, d by 1/(μ+r) so that μ=CFR/d and r=(1/d)-μ

**Diagnosis and treatment of TB**

In the baseline model the process of TB diagnosis and treatment are modelled as a simplification of the TB programme in South Africa. Individuals may be screened for TB symptoms at rates which depend on true TB and HIV status. It is assumed that those without TB will present at a lower rate while those with HIV will be screened more frequently as part of HIV care. Those identified as possible TB cases will be diagnosed with some probability (based on the assumed specificity and sensitivity of the diagnostic algorithm in South Africa (assumed to be an initial Xpert test).

Rates of screening (which implicitly include the specificity of the screening algorithm) are included in the fitting process. We allow three parameters, *k* (the screening rate in HIV uninfected TB cases), *k_pos_* (a scaling factor for the screening rate in HIV infected individuals) and *k_health_* (a scaling factor for the screening rate in individuals without TB) to vary.

The assumed sensitivity and specificity of diagnosis are given in table 2 in the main text.

Information on ILTFU, the gap between positive diagnosis and treatment initiation is limited. Based on a systematic review published in 2014 ([16](#_ENREF_16)) and data from the Xtend study ([17](#_ENREF_17)) we assumed that 17% of those with a positive diagnosis did not initiate treatment. This is parameterised in the model as the proportion of those diagnosed with TB who initiate treatment, *κ* = 100-17 = 83%.

For simplicity, individuals who start treatment are divided into those who are successfully treated (completed + cured) and those who are not. Treatment success was assumed to be independent of HIV status. Assumed values of treatment success are shown in table A.4 and are assumed to remain at current levels in the future.

| **Year** | **Treatment success (completed + cured) (%)** |
| --- | --- |
| 2006 | 70.0 |
| 2007 | 68.8 |
| 2008 | 71.3 |
| 2009 | 72.3 |
| 2010 | 70.8 |
| 2011 | 75.4 |
| 2012 | 76.1 |
| 2013 | 77.9 |
| 2014 | 77.5 |

**Table A.4.** Treatment success for DS-TB cases, *τ*. WHO TB database ([18](#_ENREF_18)).

**A.2.2 HIV model**

The HIV component of the model is similar in structure to AIM (the HIV epidemiology module of Spectrum ([19](#_ENREF_19))). The HIV infected population is divided by CD4 cell count (>500, 350-500, 250-349, 200-249, 100-199, 50-99, <50), and time since ART initiation (no ART, <6 months, 6-12 months, >12 months).

Age stratified HIV incidence, *h_i_* is an external input to the model and is based on UNAIDS estimates for South Africa. New infections are assigned to CD4 categories, *j* based on the age specific distribution of new infections, γ*_i,j_*. It is assumed that HIV incidence is unaffected by TB status. Individuals not on ART progress through CD4 categories at age (*i*) and CD4 (*j*) specific rates, *ε_i,j_*, calculated from estimated durations of CD4 stage. Those with HIV, but not on ART, are subject to HIV-associated mortality, *μH_i,j_* which is also assumed to be age and CD4 specific. Values for these parameters are given in table A.5.

Historical ART coverage is based on estimates of the number in need of ART (based on eligibility criteria) and number on ART. These values are used to calculate the percentage of the eligible population who should be on ART by age and calendar time. This percentage coverage is used in the model, together with the time dependent CD4 threshold for ART initiation, to calculate the number of people who should be on ART by age. Future ART coverage is assumed to increase in line with UNAIDS projections.

The number of people of age *a* who should be started on ART is calculated as:

$N_{start}=N_{should}-N_{on}+N_{deaths}$

That is, the number who start ART (*N_start_*) is the difference between the number who should be on ART (*N_should_*) and the number who are currently on ART (*N_on_*) plus the number who will die while on ART in the current time step (*N_deaths_*).

Those starting ART are distributed among the eligible CD4 categories based on the approach used in AIM. This depends on the proportion of the eligible (but not yet on ART) population that are in each CD4 category and the proportion of deaths among those eligible but not on ART occurring in each CD4 category.

$$P_{i,j}=\left( \frac{N_{i,j}}{\sum_{j\in e} N_{i,j}}+\frac{D_{i,j}}{\sum_{j\in e} D_{i,j}} \right)/2$$

Where *P_i,j_* is the proportion of new ART initiations of age *i* who are from CD4 category *j*, *N_i,j_* is the number of individuals of age *i* who are in CD4 category *j* and not yet on ART, and *D_i,j_* is the number of deaths occurring among those age *i* who are in CD4 category *j* and not yet on ART. Summations are over the set of CD4 categories, *e* that are below the eligibility threshold for starting ART.

Mortality rates for HIV infected individuals on ART (*μA_i,j,l_*) vary by age, CD4 at initiation, time on ART and sex and are shown in tables A.6 and A.7. As the model does not account for sex, the on-ART mortality rates are weighted by the number of males and females on ART used as input to the model.

In addition to the HIV specific TB model parameters in table A.3 the risks of developing TB are affected by HIV in a CD4 dependent manner. Parameters modified in this way are: proportion developing primary disease following first infection; rate of reactivation; the protection against disease following re-infection due to previous infection.

For each parameter *x_i_*, the CD4 dependent values are given by:

$$x_{i,j}^{H}=x_{i}{RR}_{1}{{RR}_{2}}^{(500-{mid}_{j})/100}$$

Where superscript *H* refers to HIV+ not on ART, *RR_1_* and *RR_2_* are parameter dependent relative risks and *mid_j_* is the midpoint of CD4 category *j* (note that the midpoint of the >500 CD4 category is defined as 500 such that *x_>500_* = *xRR_1_*). The proportion developing primary disease is capped at 1.

ART reverses the impact of HIV on the proportion developing primary disease, the rate of reactivation and the protection provided by prior infection. It also reduces the TB mortality rate compared to the rate in HIV positive individuals not on ART.

For the proportion developing disease, reactivation and mortality the adjusted parameters are given by:

$${x^{A}}_{i,j,l}=max\left( {x^{H}}_{i,j}\left( 1-{ART}_{l} \right),x_{i} \right)$$

where superscript *A* refers to HIV+ on ART, *j* is CD4 at time of ART initiation (not CD4 progression is not modelled in those on ART) and *l* is time on ART. *ART_l_* is the protective effect of ART by time on ART (*ART_l_* can differ for disease and mortality) and the *max* ensures that ART does not reduce the risks below those experienced by HIV uninfected individuals of age (*x_i_*).

ART increases the protection provided by prior infection (which is not age dependent) and is implemented as follows:

$${x^{A}}_{j,l}=min\left( {1-(1-x^{H}}_{j}\left( 1-{ART}_{l} \right),x \right)$$

|  | **Age** | | | | | | |
| --- | --- | --- | --- | --- | --- | --- | --- |
| **CD4** | **0-4** | **5-9** | **10-14** | **15-24** | **25-34** | **35-44** | **45+** |
| Distribution of new HIV infections (%), *γ* | | | | | | | |
| **>500** | 64.3 | 64.3 | 64.3 | 64.3 | 60.7 | 58.5 | 55.2 |
| **350-500** | 35.7 | 35.7 | 35.7 | 35.7 | 39.3 | 41.5 | 44.8 |
| **250-349** | 0 | 0 | 0 | 0 | 0 | 0 | 0 |
| **200-249** | 0 | 0 | 0 | 0 | 0 | 0 | 0 |
| **100-199** | 0 | 0 | 0 | 0 | 0 | 0 | 0 |
| **50-99** | 0 | 0 | 0 | 0 | 0 | 0 | 0 |
| **<50** | 0 | 0 | 0 | 0 | 0 | 0 | 0 |
| Rates of progression through CD4 categories (years^-1^), *ε* | | | | | | | |
| **>500** | 0.298 | 0.298 | 0.298 | 0.117 | 0.147 | 0.183 | 0.213 |
| **350-500** | 0.239 | 0.239 | 0.239 | 0.223 | 0.240 | 0.355 | 0.535 |
| **250-349** | 0.183 | 0.183 | 0.183 | 0.294 | 0.452 | 0.581 | 0.855 |
| **200-249** | 0.183 | 0.183 | 0.183 | 0.508 | 1.087 | 1.250 | 1.818 |
| **100-199** | 0.130 | 0.130 | 0.130 | 0.214 | 0.637 | 0.676 | 0.952 |
| **50-99** | 0.130 | 0.130 | 0.130 | 0.348 | 1.449 | 1.449 | 2.00 |
| HIV mortality rates (per year) in the absence of ART, *μH* | | | | | | | |
| **>500** | 0.312 | 0.039 | 0.039 | 0.005 | 0.004 | 0.005 | 0.005 |
| **350-500** | 0.382 | 0.048 | 0.048 | 0.011 | 0.01 | 0.013 | 0.013 |
| **250-349** | 0.466 | 0.058 | 0.058 | 0.026 | 0.026 | 0.036 | 0.032 |
| **200-249** | 0.466 | 0.058 | 0.058 | 0.061 | 0.069 | 0.096 | 0.08 |
| **100-199** | 0.569 | 0.071 | 0.071 | 0.139 | 0.185 | 0.258 | 0.203 |
| **50-99** | 0.569 | 0.071 | 0.071 | 0.321 | 0.499 | 0.691 | 0.513 |
| **<50** | 0.569 | 0.071 | 0.071 | 0.737 | 1.342 | 1.851 | 1.295 |

**Table A.5.** HIV model parameters

|  | **Age** | | | | | | |
| --- | --- | --- | --- | --- | --- | --- | --- |
| **CD4** | **0-4** | **5-9** | **10-14** | **15-24** | **25-34** | **35-44** | **45+** |
| 0-6 months on ART | | | | | | | |
| **>500** | 0.0568 | 0.0071 | 0.0067 | 0.0050 | 0.0035 | 0.0050 | 0.0050 |
| **350-500** | 0.2567 | 0.0321 | 0.0301 | 0.0115 | 0.0095 | 0.0134 | 0.0126 |
| **250-349** | 0.1945 | 0.0241 | 0.0228 | 0.0264 | 0.0256 | 0.0359 | 0.0319 |
| **200-249** | 0.1945 | 0.0241 | 0.0228 | 0.0607 | 0.0563 | 0.0587 | 0.0594 |
| **100-199** | 0.6814 | 0.0852 | 0.0799 | 0.1113 | 0.0929 | 0.0980 | 0.1039 |
| **50-99** | 0.6814 | 0.0852 | 0.0799 | 0.1810 | 0.1525 | 0.1619 | 0.1762 |
| **<50** | 0.6814 | 0.0852 | 0.0799 | 0.3974 | 0.3373 | 0.3605 | 0.4009 |
| 7-12 months on ART | | | | | | | |
| **>500** | 0.1501 | 0.0188 | 0.0088 | 0.0050 | 0.0035 | 0.0050 | 0.0050 |
| **350-500** | 0.2148 | 0.0269 | 0.0125 | 0.0115 | 0.0095 | 0.0134 | 0.0126 |
| **250-349** | 0.2039 | 0.0269 | 0.0119 | 0.0244 | 0.0256 | 0.0323 | 0.0319 |
| **200-249** | 0.2039 | 0.0269 | 0.0119 | 0.0258 | 0.0332 | 0.0341 | 0.0451 |
| **100-199** | 0.3850 | 0.0481 | 0.0225 | 0.0323 | 0.0417 | 0.0433 | 0.0584 |
| **50-99** | 0.3850 | 0.0481 | 0.0225 | 0.0405 | 0.0523 | 0.0548 | 0.0751 |
| **<50** | 0.3850 | 0.0481 | 0.0225 | 0.0583 | 0.0753 | 0.0797 | 0.1112 |
| >12 months on ART | | | | | | | |
| **>500** | 0.0636 | 0.0080 | 0.0037 | 0.0050 | 0.0035 | 0.0050 | 0.0050 |
| **350-500** | 0.0910 | 0.0114 | 0.0053 | 0.0086 | 0.0095 | 0.0101 | 0.0102 |
| **250-349** | 0.0864 | 0.0108 | 0.0050 | 0.0092 | 0.0117 | 0.0109 | 0.0114 |
| **200-249** | 0.0864 | 0.0108 | 0.0050 | 0.0098 | 0.0125 | 0.0118 | 0.0127 |
| **100-199** | 0.1632 | 0.0204 | 0.0095 | 0.0129 | 0.0165 | 0.0161 | 0.0190 |
| **50-99** | 0.1632 | 0.0204 | 0.0095 | 0.0168 | 0.0216 | 0.0216 | 0.0268 |
| **<50** | 0.1632 | 0.0204 | 0.0095 | 0.0251 | 0.0333 | 0.0333 | 0.0438 |

**Table A.6.** ART mortality parameters, *μA*, male (per year)

|  | **Age** | | | | | | |
| --- | --- | --- | --- | --- | --- | --- | --- |
| **CD4** | **0-4** | **5-9** | **10-14** | **15-24** | **25-34** | **35-44** | **45+** |
| 0-6 months on ART | | | | | | | |
| **>500** | 0.0680 | 0.0085 | 0.0080 | 0.0050 | 0.0035 | 0.0050 | 0.0050 |
| **350-500** | 0.3074 | 0.0384 | 0.0360 | 0.0115 | 0.0095 | 0.0134 | 0.0126 |
| **250-349** | 0.2329 | 0.0291 | 0.0273 | 0.0264 | 0.0256 | 0.0359 | 0.0319 |
| **200-249** | 0.2329 | 0.0291 | 0.0273 | 0.0529 | 0.0431 | 0.0445 | 0.0433 |
| **100-199** | 0.8160 | 0.1020 | 0.0957 | 0.0866 | 0.0719 | 0.0754 | 0.0783 |
| **50-99** | 0.8160 | 0.1020 | 0.0957 | 0.1415 | 0.1187 | 0.0126 | 0.1352 |
| **<50** | 0.8160 | 0.1020 | 0.0957 | 0.3118 | 0.2641 | 0.2819 | 0.3120 |
| 7-12 months on ART | | | | | | | |
| **>500** | 0.1430 | 0.0179 | 0.0083 | 0.0050 | 0.0035 | 0.0050 | 0.0050 |
| **350-500** | 0.2046 | 0.0256 | 0.0119 | 0.0115 | 0.0095 | 0.0134 | 0.0126 |
| **250-349** | 0.1942 | 0.0243 | 0.0113 | 0.0183 | 0.0235 | 0.0237 | 0.0299 |
| **200-249** | 0.1942 | 0.0243 | 0.0113 | 0.0193 | 0.0248 | 0.0251 | 0.0320 |
| **100-199** | 0.3667 | 0.0458 | 0.0214 | 0.0245 | 0.0315 | 0.0323 | 0.0425 |
| **50-99** | 0.3667 | 0.0458 | 0.0214 | 0.0309 | 0.0399 | 0.0414 | 0.0556 |
| **<50** | 0.3667 | 0.0458 | 0.0214 | 0.0449 | 0.0580 | 0.0610 | 0.0840 |
| >12 months on ART | | | | | | | |
| **>500** | 0.0606 | 0.0076 | 0.0035 | 0.0050 | 0.0035 | 0.0050 | 0.0042 |
| **350-500** | 0.0867 | 0.0108 | 0.0051 | 0.0058 | 0.0074 | 0.0062 | 0.0046 |
| **250-349** | 0.0823 | 0.0103 | 0.0050 | 0.0063 | 0.0080 | 0.0069 | 0.0055 |
| **200-249** | 0.0823 | 0.0103 | 0.0048 | 0.0068 | 0.0086 | 0.0076 | 0.0065 |
| **100-199** | 0.1554 | 0.0194 | 0.0091 | 0.0092 | 0.0118 | 0.0110 | 0.0115 |
| **50-99** | 0.1554 | 0.0194 | 0.0091 | 0.0123 | 0.0157 | 0.0152 | 0.0176 |
| **<50** | 0.1554 | 0.0194 | 0.0091 | 0.0188 | 0.0242 | 0.0244 | 0.0310 |

**Table A.7.** ART mortality parameters, *μA*, female (per year)

**A.2.3 Demographic model**

The model is age structured in 5-year age bins (0-4,5-9, ....75-80) and a single bin representing those 80+ (17 age groups in total). Demographic parameters are taken from the UN population Division ([20](#_ENREF_20)).

Births are modelled using the crude birth rate (/1000 population) and are added into the susceptible population (age 0-4) as a fixed event at the start of each year. Aging is also modelled as a discrete event following the method of Schenzle ([21](#_ENREF_21)).

Migration, *m_i_* is modelled as a continuous process based on estimated net numbers of migrants by age, *i*, and calendar year. Migration does not depend on disease state (TB or HIV). Instead, the total number of migrants in a given age group is divided between disease states based on the relative size of compartments.

Deaths are modelled using mortality rates derived from UN population division life tables. Because these mortality rates include deaths due to HIV and TB it is necessary to correct for disease induced mortality in the model to derive the age specific background mortality rate, *μ_i_*. This is achieved by reducing the UN estimated mortality rates at each time point by the rate of disease (HIV and TB) mortality. In the same way, the HIV mortality rates include deaths in HIV infected individuals due to TB and must also be corrected to avoid double counting of these deaths in the model. This correction is applied up to 2015 after which the reduction in background mortality is fixed at the 2015 levels. This allows changes in future TB burden to be reflected in the overall mortality. Figure A.6 shows the comparison of the model population to UN population estimates

**A.2.4 Equations**

The model is implemented as a set of differential equations in the C programming language as a compiled DLL and solved using the deSolve package in R.

The following equations describe the baseline model for HIV uninfected individuals. As mentioned above, aging and births are modelled as a fixed event at the start of each year. Subscript *i* indicates age.

**HIV uninfected**

$$\frac{dS_{i}}{dt}=-\lambda S_{i}-h_{i}S_{i}-\mu_{i}S_{i}+\frac{m_{i}S_{i}}{N_{i}}$$

$$\frac{dL_{i}}{dt}=\lambda\left( 1-a_{i} \right)\left( S_{i}+C_{i} \right)+\lambda a_{i}pC_{i}-\left( v_{i}+\lambda a_{i}\left( 1-p \right) \right)L_{i}+r_{I}I_{i}+r_{N}N_{i}-\delta_{F}\kappa\tau L_{i}+\delta_{T}\kappa\tau(N_{i}+I_{i})-h_{i}L_{i}-\mu_{i}L_{i}+\frac{m_{i}L_{i}}{N_{i}}$$

$$\frac{{dN}_{i}}{dt}=\lambda a_{i}\left( 1-\sigma_{i} \right)\left( S_{i}+\left( 1-p \right)\left( L_{i}+C_{i} \right) \right)+v_{i}\left( 1-\sigma_{i} \right)L_{i}-\left( \vartheta+r_{N}+{\mu N}_{i}+\mu_{i}+h_{i} \right)N_{i}-\frac{m_{i}N_{i}}{N_{i}}-\delta_{T}\kappa\tau N_{i}$$

$$\frac{{dIsn}_{i}}{dt}=\lambda a_{i}\sigma_{i}\left( S_{i}+\left( 1-p \right)\left( L_{i}+C_{i} \right) \right)+v_{i}\sigma_{i}L_{i}+\vartheta N_{i}-\left( r_{I}+{\mu I}_{i}+\mu_{i}+h_{i} \right)I_{i}-\frac{m_{i}I_{i}}{N_{i}}-\delta_{T}\kappa\tau{Isn}_{i}$$

$$\frac{{dC}_{i}}{dt}=\delta_{F}\kappa\tau{Lsn}_{i}-\lambda\left( 1-a_{i} \right)C_{i}-\lambda a_{i}\left( 1-p \right)C_{i}-h_{i}C_{i}-\mu_{i}C_{i}+\frac{m_{i}C_{i}}{N_{i}}$$

**HIV infected, not on ART**

The equations for HIV infected individuals not on ART are similar to those described above, but with additional terms describing acquisition of HIV (*h_i_*), progression through CD4 states *ε_i,j_*, ART initiation (*ART_i,j_*) and HIV associated mortality (*μH_i,j_*) as illustrated in the equation for the susceptible population below. In addition, HIV specific parameters are used as described previously.

$$\frac{d{SH}_{i,j}}{dt}=-\lambda{SH}_{i,j}+h_{i}\gamma_{i,j}S_{i}-\varepsilon_{i,j}{SH}_{i,j}+\varepsilon_{i,j-1}{SH}_{i,j-1}-(\mu_{i}+{\mu H}_{i,j}+{ART}_{i,j}){SH}_{i,j}+m_{i}{SH}_{i,j}/N_{i}$$

**HIV infected, on ART**

The equations for HIV infected individuals on ART include additional terms describing initiation of ART (*ART_i,j_*), progression through time on ART (*η_i,j,l_*) HIV associated mortality while on ART (*μA_i,j,l_*) as illustrated in the equation for the susceptible population below. In addition, ART adjusted parameters are used as described previously.

$$\frac{d{SA}_{i,j,l}}{dt}=-\lambda{SA}_{i,j,l}-\eta_{i,j,l}{SA}_{i,jl}+\eta_{i,j,l-1}{SA}_{i,j,l-1}-(\mu_{i}+{\mu_{A}}_{i,j,l}){SA}_{i,j,l}+{ART}_{i,j}{SH}_{i,j}+m_{i}{SA}_{i,j,l}/N_{i}$$

**Force of infection**

$\lambda=\beta(\sum_{i} I_{i}+\sum_{i,j} {IH}_{i,j}+\sum_{i,j,l} {IA}_{i,j,l}+\omega(\sum_{i} {Nsn}_{i}+\sum_{i,j} {NsnH}_{i,j}+\sum_{i,j,l} {NsnA}_{i,j,l}))/N$

**A.2.5 Model calibration**

The model was calibrated using a sampling-importance-resampling (SIR) approach ([22](#_ENREF_22), [23](#_ENREF_23)). 100,000 parameter sets were drawn from the prior distributions given in table A.2, the model run for each set and the likelihood calculated. The parameter sets were then resampled (with replacement) to generate a sample of size 10,000 using the likelihood as importance weights. The model was calibrated to the WHO estimated TB incidence and mortality (rate/100,000), TB notifications, the proportion of incident TB in HIV infected individuals and the proportion of TB in children (<15 years of age) in 2015 (13.2% (6.4-25.3%)) in South Africa. All calibration data were assumed to be normally distributed with median and 95% CI as in table A.8. In the absence of other data, for notifications we assumed a 95% CI extending +/- 10% of the reported value to allow for under or over reporting.

|  | **Notifications (/100k)** | **Incidence (/100k)** | | **Mortality (/100k)** |
| --- | --- | --- | --- | --- |
| **Year** | **All** | **All** | **HIV+** | **All** |
| 1990 | 219 | 313 (203-447) | 14 (9-20) | 52 (33-72) |
| 1991 | 206 | 309 (200-442) | 28 (18-41) | 50 (31-66) |
| 1992 | 214 | 299 (194-427 | 48 (30-70) | 45 (27-59) |
| 1993 | 227 | 290 (188-414) | 71 (45-103) | 41 (25-55) |
| 1994 | 223 | 289 (187-412) | 95 (60-138) | 40 (25-56) |
| 1995 | 178 | 302 (196-432) | 121 (77-175) | 42 (30-61) |
| 1996 | 259 | 333 (216-476) | 152 (96-220) | 54 (40-73) |
| 1997 | 293 | 379 (246-542) | 190 (120-275) | 75 (56-98) |
| 1998 | 326 | 439 (284-627) | 233 (148-338) | 99 (73-126) |
| 1999 | 335 | 509 (330-727) | 280 (177-406) | 124 (93-165) |
| 2000 | 337 | 585 (379-836) | 349 (221-506) | 158 (116-203) |
| 2001 | 325 | 666 (431-951) | 403 (255-584) | 189 (141-235) |
| 2002 | 465 | 746 (482-1065) | 455 (288-660) | 217 (165-268) |
| 2003 | 484 | 820 (530-1171) | 502 (318-728) | 241 (183-288) |
| 2004 | 561 | 883 (571-1261) | 542 (343-786) | 257 (196-309) |
| 2005 | 559 | 932 (603-1331) | 571 (361-828) | 263 (197-326) |
| 2006 | 618 | 963 (623-1375) | 588 (372-853) | 260 (196-325) |
| 2007 | 635 | 977 (632-1395) | 595 (376-863) | 248 (187-302) |
| 2008 | 692 | 977 (717-1276) | 593 (375-860) | 237 (177-298) |
| 2009 | 706 | 967 (728-1238) | 585 (370-848) | 226 (165-289) |
| 2010 | 687 | 948 (710-1219) | 571 (361-829) | 214 (155-282) |
| 2011 | 694 | 922 (712-1159) | 603 (387-867) | 194 (142-254) |
| 2012 | 613 | 892 (639-1186) | 570 (366-820) | 176 (128-233) |
| 2013 | 585 | 860 (612-1149) | 530 (340-761) | 169 (117-229) |
| 2014 | 567 | 834 (539-1114) | 508 (236-729) | 169 (117-229) |
| 2015 | 527 | 834 (539-1190) | 473 (303-680) | 169 (117-229) |

**Table A.8**. Calibration data. Notifications, incidence and mortality were taken from the WHO notifications ([24](#_ENREF_24)) and burden databases ([25](#_ENREF_25)).

**A.3 Additional transmission model outputs**

**A.3.1 Baseline fit**

**
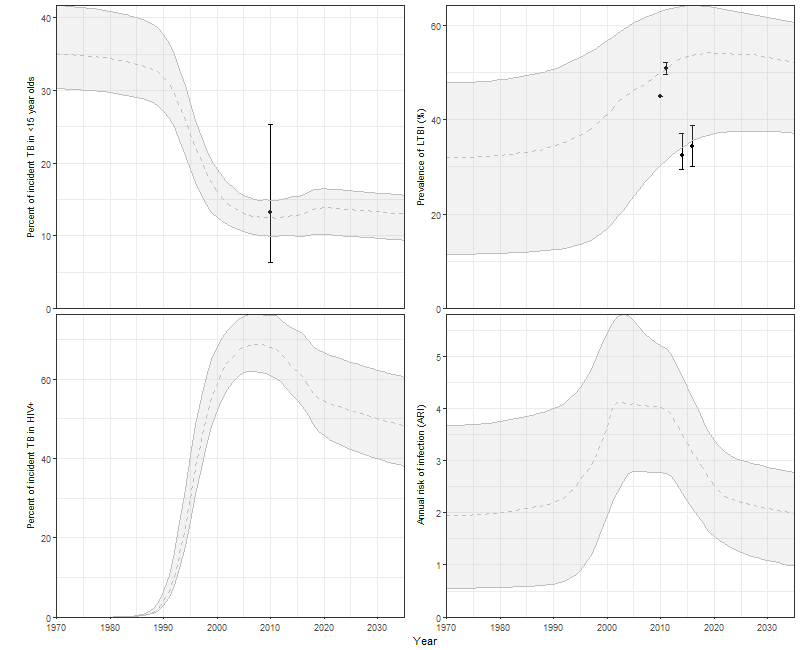
**

**Figure A.4.** Additional baseline TB outputs. Grey shaded areas show model outputs (dashed line = median, ribbon = 95% CI). Black points and error bars show data. The model was calibrated to the percentage of TB in children but not to the prevalence of LTBI.

**
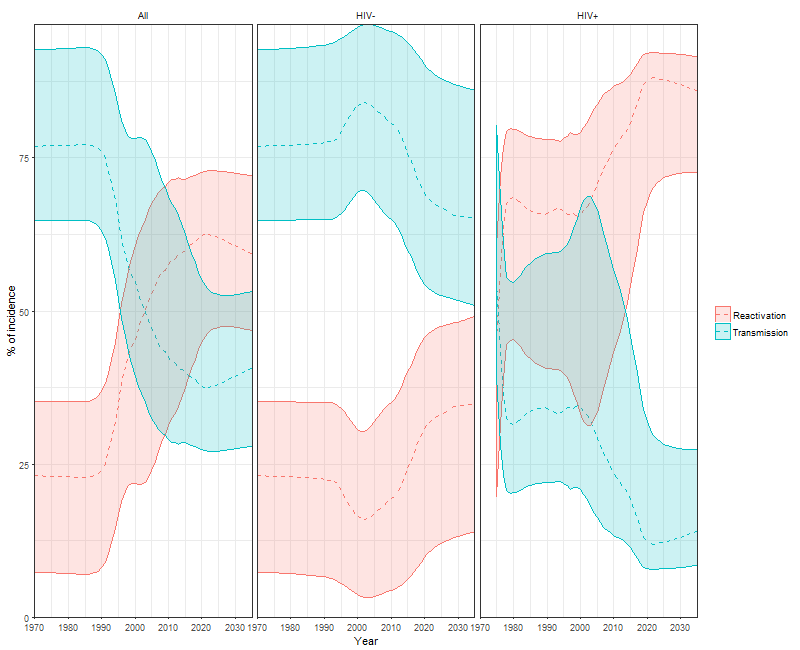
**

**Figure A.5.** Proportion of incident TB due to reactivation vs transmission disaggregated by HIV status.

**
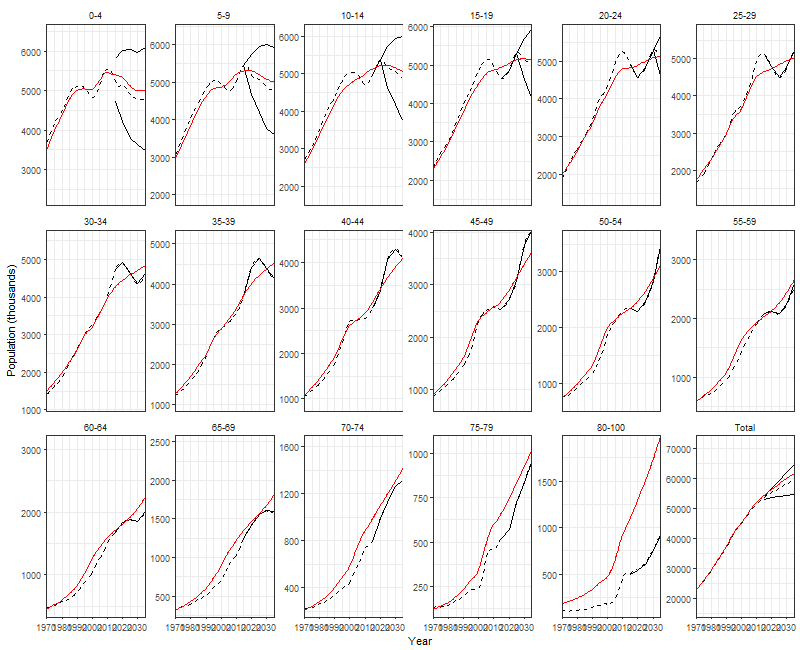
**

**Figure A.6. Demographic fit.** UN Population division projections are shown in black. Model outputs in red. Panels show population (in thousands) by 5-year age groups as indicated

**
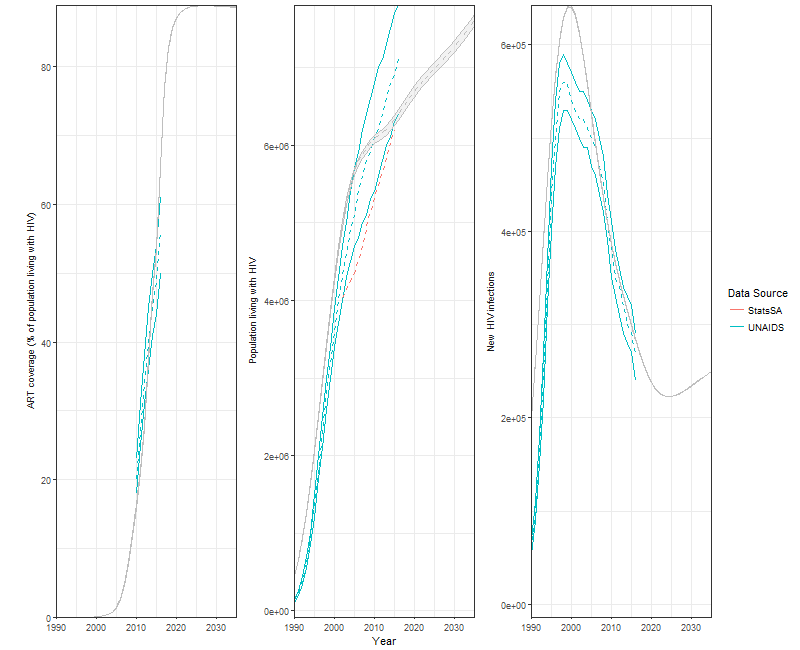
**

**Figure A.7. HIV model outputs.** Left panel: ART coverage; centre panel: HIV positive population; right panel: new HIV infections. Data is shown by coloured lines (blue = UNAIDS, red = Statistics SA), dashed lines = mid-point estimate, solid lines indicate range. Model outputs are shown by grey shaded areas.

**A.3.2 Varying screening coverage**

Table A.9. shows the results of the transmission model for different screening coverages.

|  | **COR** | **IGRA** | | **Ratio (IGRA:COR)** |
| --- | --- | --- | --- | --- |
| **Coverage** | **Reduction in TB incidence in 2020** | | | |
| 5% | 0.9 (0.6-1.2) | 1.0 (0.7-1.3) | | 1.1 |
| 10% | 1.7 (1.2-2.5) | 1.9 (1.3-2.7) | | 1.1 |
| 20% | 3.5 (2.3-4.8) | 3.8 (2.6-5.2) | | 1.1 |
| 30% | 5.1 (3.5-7.2) | 5.6 (3.9-7.7) | | 1.1 |
|  | **Reduction in TB incidence in 2035** | |  | |
| 5% | 4.0 (2.9-5.5) | 9.6 (7.1-12.9) | | 2.4 |
| 10% | 7.7 (5.6-10.6) | 17.6 (13.3-23.2) | | 2.3 |
| 20% | 14.5 (10.6-19.4) | 30.0 (23.5-38.2) | | 2.1 |
| 30% | 20.4 (15.1-27.0) | 38.8 (31.2-48.2) | | 1.9 |
|  | **Cases averted in 2020 (thousands)** | | | |
| 5% | 2.2 (1.4-3.4) | 2.5 (1.5-3.8) | | 1.1 |
| 10% | 4.4 (2.8-6.7) | 4.9 (3.1-7.5) | | 1.1 |
| 20% | 8.8 (5.5-13.3) | 9.6 (6.1-14.8) | | 1.1 |
| 30% | 13.0 (8.2-19.7) | 14.2 (9.0-21.8) | | 1.1 |
|  | **Cases averted in 2020-2035 (thousands)** | | | |
| 5% | 94.0 (60.0-151.2) | 186.7 (115.8-300.9) | | 2.0 |
| 10% | 182.5 (116.8-292.2) | 351.0 (219.9-560.9) | | 1.9 |
| 20% | 344.4 (222.3-545.1) | 624.7 (396.6-981.7) | | 1.8 |
| 30% | 488.8 (317.4-767.3) | 839.6 (541.3-1295.7) | | 1.7 |
|  | **PT given per year (millions)** | | | |
| 5% | 0.25 (0.18-0.32) | 0.77 (0.57-0.91) | | 3.1 |
| 10% | 0.50 (0.37-0.65) | 1.5 (1.1-1.8) | | 3.1 |
| 20% | 1.0 (0.74-1.3) | 3.1 (2.3-3.6) | | 3.1 |
| 30% | 1.5 (1.1-1.9) | 4.6 (3.4-5.3) | | 3.1 |
|  | **NNT to avert one case in 2020** | | | |
| 5% | 103 (62-173) | 290 (201-430) | | 2.8 |
| 10% | 104 (62-174) | 292 (203-432) | | 2.8 |
| 20% | 105 (63-176) | 296 (206-438) | | 2.8 |
| 30% | 107 (64-178) | 301 (210-433) | | 2.8 |
|  | **NNT to avert one case 2020-2035** | | | |
| 5% | 42 (25-68) | 65 (43-98) | | 1.5 |
| 10% | 44 (26-70) | 68 (46-103) | | 1.6 |
| 20% | 46 (28-73) | 76 (52-113) | | 1.7 |
| 30% | 49 (29-77) | 85 (59-123) | | 1.7 |

**Table A.9**. Results of the transmission model for different screening coverages.

**A.3.3 Sensitivity Analysis**

Figure A.8 shows partial rank correlation coefficients (PRCCs) between the model output and the model parameters. Results are for the COR 60 strategy, assuming 3HP cures and that individuals can receive multiple rounds of 3HP. Red bars indicate PRCCs for the % reduction in incidence, blue bars those for the cumulative doses of 3HP per case averted (NNT), both measured in 2035.

The impact of the COR strategy shows a moderate positive correlation with the sensitivity of COR among progressors (SE_I_COR) and a very strong positive correlation with the adherence to and efficacy of 3HP (adh_3HP and eff_3HP respectively). These parameters are negatively correlated with the NNT (which declines as the impact increases assuming the number treated remains constant).

Interestingly, the impact of the COR strategy is strongly negatively correlated with the specificity of the COR (SP_COR) (the impact is increased as the specificity is reduced). Reducing the specificity leads to increased treatment with 3HP of latently infected individuals who are not at immediate risk of progression to TB. However, these individuals do have a non-zero lifetime risk of TB disease which is reduced assuming 3HP “cures” LTBI. As a result, the impact is increased as the specificity is reduced. However, the NNT also increases as the specificity is reduced; the increase in the number given 3HP outweighs the increase in the reduction in TB incidence.

**
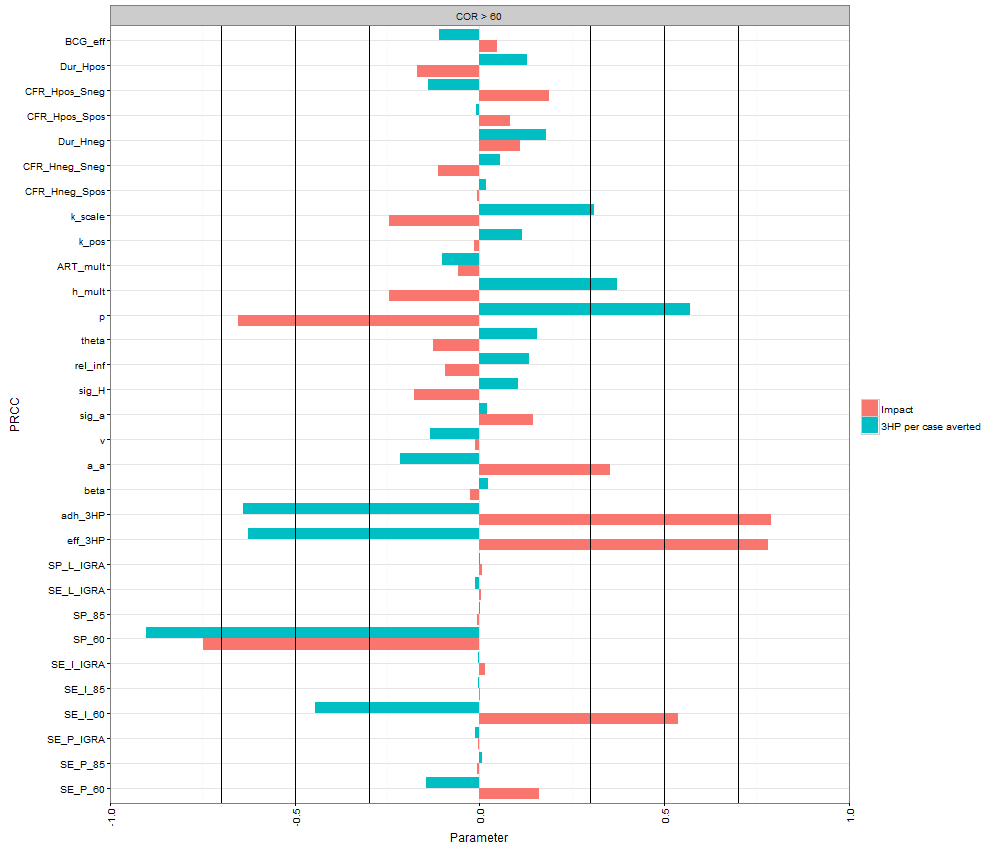
**

**Figure A.8. Partial rank correlation coefficients for the reduction in incidence in 2035.** Positive values indicate a positive correlation, negative values a negative correlation. Red dots are for COR > 60 strategy, blue dots for IGRA.

In addition to looking at the sensitivity to individual parameters, it is useful to look at how the model outputs correlate with intermediate model variables. These are determined by combinations of parameters and maybe more easily interpretable and measurable in practice.

Figure A.9 shows the relationship between the predicted reduction in incidence in 2035 using the COR strategy and several intermediate model outputs (calculated in 2020, the year the intervention is introduced). These indicate that the impact increases as the incidence of TB, the annual risk of infection (ARI) and the proportion of TB in children under 15 years of age increases. All these variables are associated with increased levels of ongoing transmission and hence a bigger potential indirect effect of preventing progression to TB on future transmission. Similarly, the impact is inversely correlated with the proportion of TB due to reactivation of prior infections, again indicating an increased effect in transmission driven epidemics, and the proportion of TB in PLWHIV. As the intervention does not target this group, an increased proportion of disease in this population reduces the potential impact. Interestingly, the impact is positively correlated with the prevalence of latent infection. This could be because of several reasons. Firstly, a high prevalence of LTBI is consistent with high levels of transmission and hence an increased impact (see above). Secondly, high levels of LTBI mean there is an increased population who can get some long-term benefit from being provided 3HP. The associations between these model variables and the NNT are shown in figure A.10. As expected these are the inverse of the associations with the intervention impact.

**
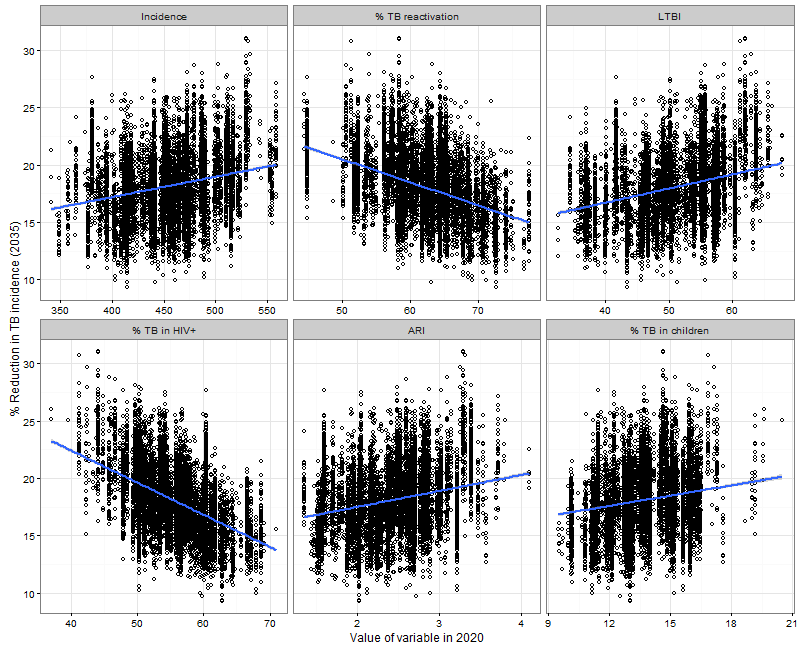
**

**Figure A.9.** Scatter plots of % reduction in incidence in 2035 as a result of COR vs value of model variable in 2020 (the year of COR introduction). Blue lines show linear regression.

**
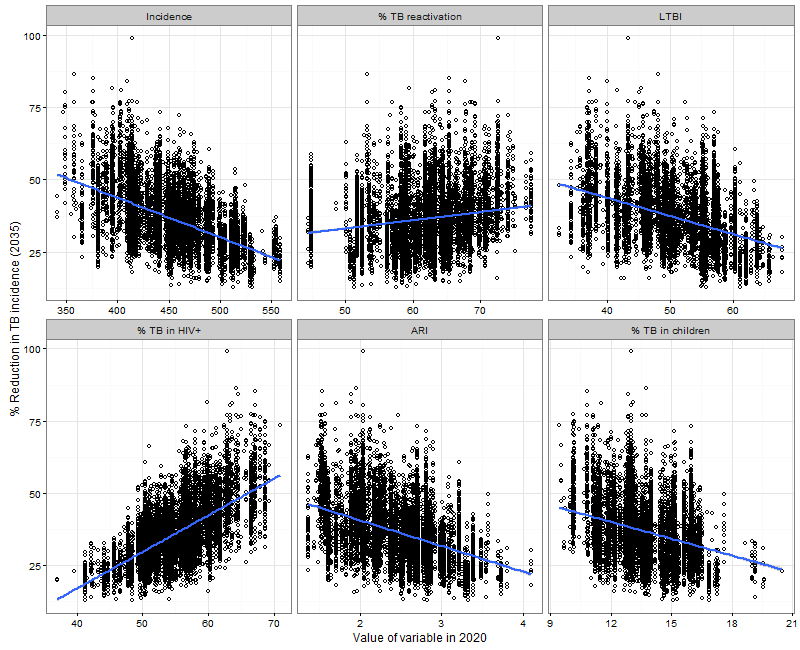
**

**Figure A.10.** Scatter plots of NNT with 3HP to avert one TB case (cumulative to 2035) as a result of COR vs value of model variable in 2020 (the year of COR introduction). Blue lines show linear regression.

**A.3.4 Scenario analysis**

Figure A.11 and table A.10 show the predicted impact of each strategy for different assumptions about the ability of 3HP to cure infection (cure vs no cure) and the number of courses of 3HP an individual can receive (repeat vs single). Figures A.12-A.15 show the number tested, treated and given 3HP and the NNT with 3HP to avert one TB case for these same assumptions. These results are discussed in more detail in the main text.

**
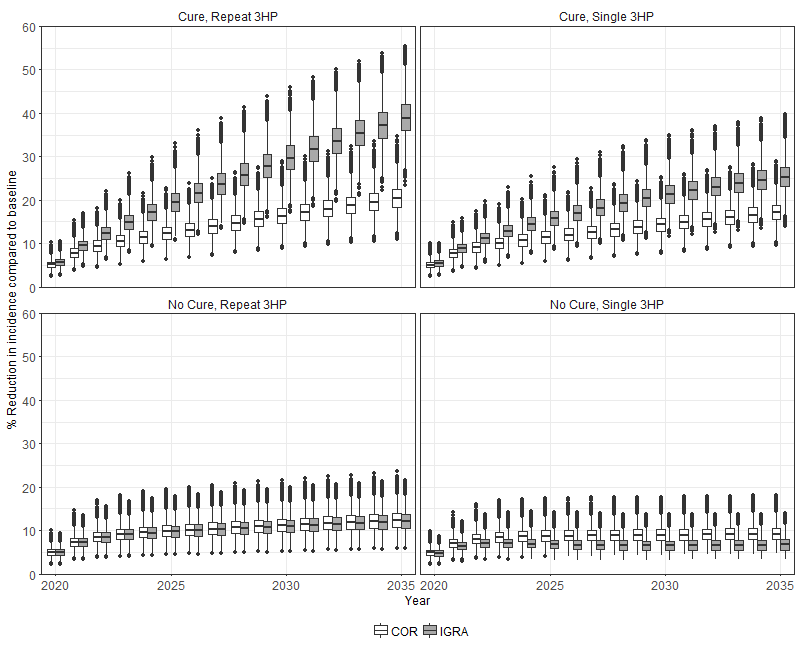
**

**Figure A.11. Predicted reduction in incidence for different cure and frequency assumptions.** Top left: 3HP cures, multiple rounds allowed (primary analysis); top right: 3HP cures, single round of 3HP; bottom left: 3HP does not cure, multiple rounds allowed; bottom right: 3HP does not cure, single round of 3HP. Shading indicates strategy. Boxes show the median and interquartile range (IQR). Whiskers show the largest values (or 1.5 IQR). Dots indicates outliers.

|  | **COR** | **IGRA** |
| --- | --- | --- |
| **Multiple, Cure** | | |
| 2020 | 5.1 (3.5-7.2) | 5.6 (3.9-7.7) |
| 2035 | 20.4 (15.2-26.9) | 38.8 (31.2-48.0) |
| **Multiple, No cure** | | |
| 2020 | 5.0 (3.4-7.0) | 5.0 (3.4-6.9) |
| 2035 | 12.3 (8.3-17.7) | 12.0 (8.1-17.3) |
| **Single, Cure** | | |
| 2020 | 5.1 (3.4-7.1) | 5.4 (3.8-7.5) |
| 2035 | 17.0 (12.8-22.5) | 25.3 (19.4-32.7) |
| **Single, No cure** |  |  |
| 2020 | 4.9 (3.3-6.9) | 4.7 (3.2-6.6) |
| 2035 | 9.2 (6.1-13.7) | 6.7 (4.3-11.5) |
| **No PT (impact due to identifying and treating prevalent TB only)** | | |
| 2020 | 0.9 (0.5-1.5) | 0.8 (0.5-1.4) |
| 2035 | 3.7 (21.-6.6) | 3.6 (21.-6.3) |

**Table A.10. % reduction in incidence in 2020 and 2035 for different scenarios.**

**
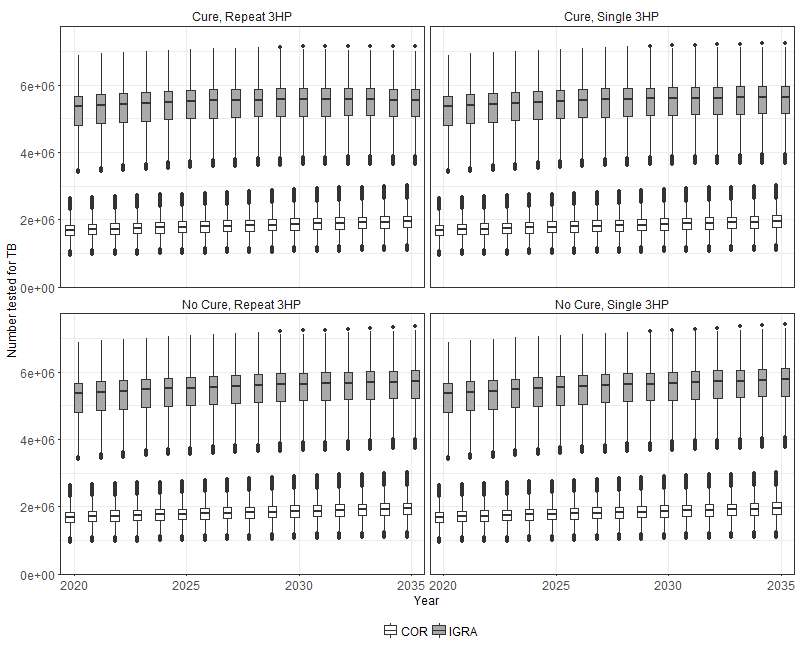
**

**Figure A.12. Number tested for TB for different cure and frequency assumptions.** Top left: 3HP cures, multiple rounds allowed (primary analysis); top right: 3HP cures, single round of 3HP; bottom left: 3HP does not cure, multiple rounds allowed; bottom right: 3HP does not cure, single round of 3HP. Shading indicates strategy. Boxes show the median and interquartile range (IQR). Whiskers show the largest values (or 1.5 IQR). Dots indicates outliers.

**
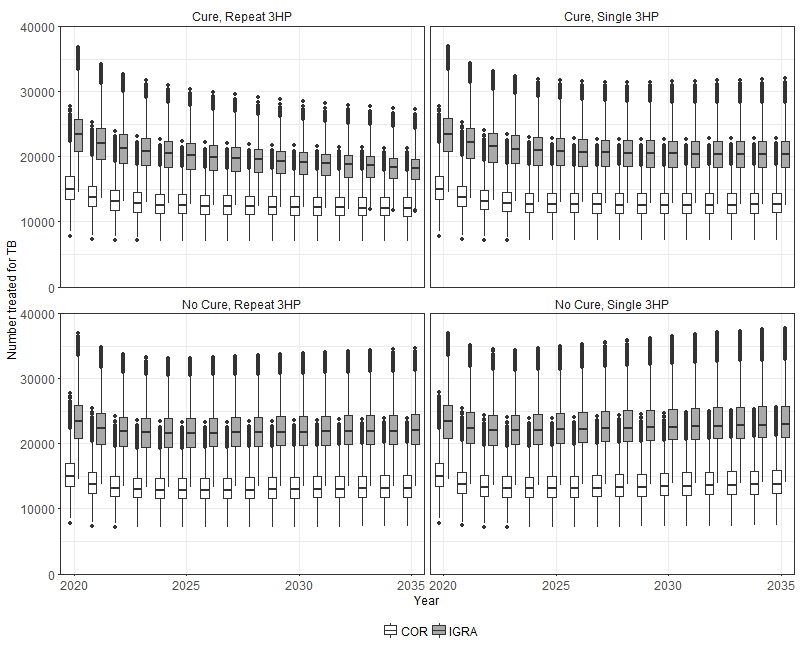
**

**Figure A.13 Number treated for TB for different cure and frequency assumptions.** Top left: 3HP cures, multiple rounds allowed (primary analysis); top right: 3HP cures, single round of 3HP; bottom left: 3HP does not cure, multiple rounds allowed; bottom right: 3HP does not cure, single round of 3HP. Shading indicates strategy. Boxes show the median and interquartile range (IQR). Whiskers show the largest values (or 1.5 IQR). Dots indicates outliers.

**
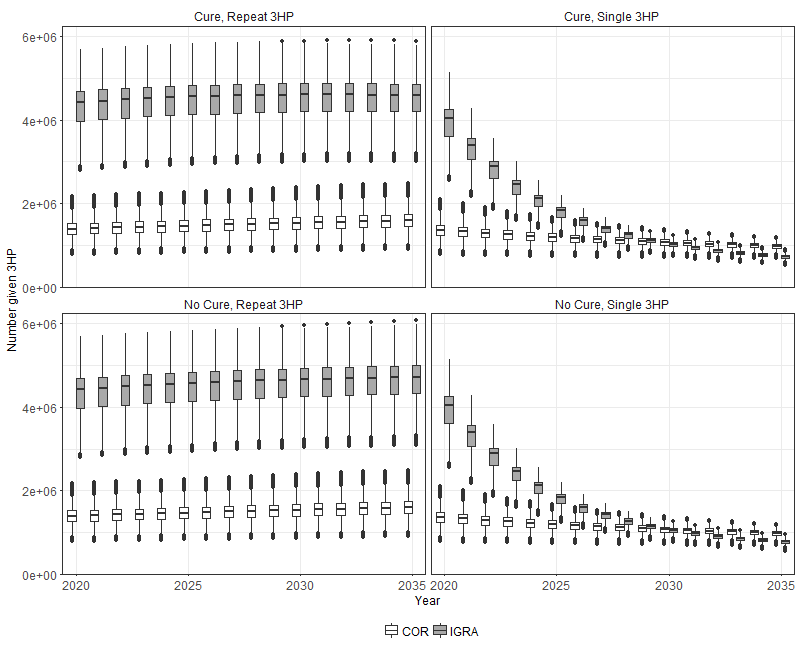
**

**Figure A.14 Number given 3HP for different cure and frequency assumptions.** Top left: 3HP cures, multiple rounds allowed (primary analysis); top right: 3HP cures, single round of 3HP; bottom left: 3HP does not cure, multiple rounds allowed; bottom right: 3HP does not cure, single round of 3HP. Shading indicates strategy. Boxes show the median and interquartile range (IQR). Whiskers show the largest values (or 1.5 IQR). Dots indicates outliers.

**
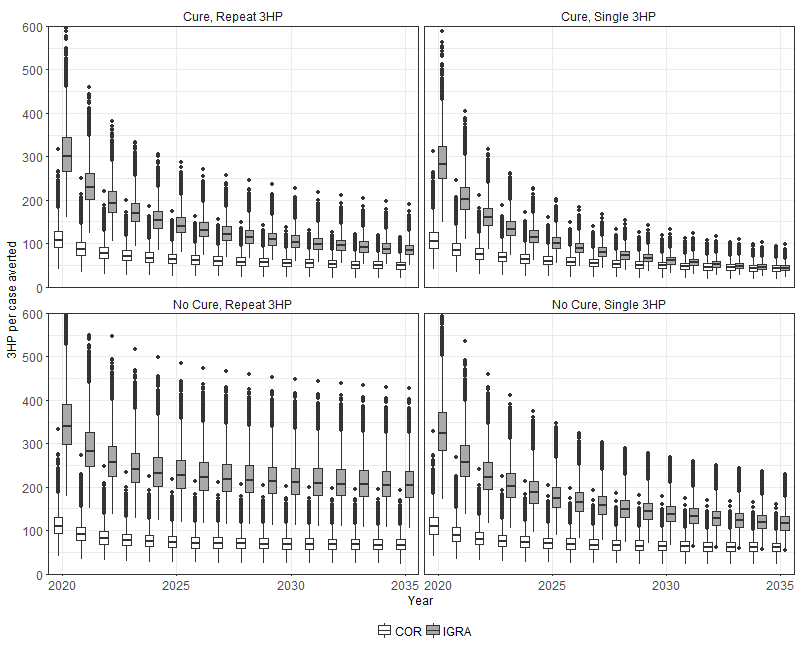
**

**Figure A.15 NNT for different cure and frequency assumptions.** Top left: 3HP cures, multiple rounds allowed (primary analysis); top right: 3HP cures, single round of 3HP; bottom left: 3HP does not cure, multiple rounds allowed; bottom right: 3HP does not cure, single round of 3HP. Shading indicates strategy. Boxes show the median and interquartile range (IQR). Whiskers show the largest values (or 1.5 IQR). Dots indicates outliers.

**References**

1. Petruccioli E, Scriba TJ, Petrone L, Hatherill M, Cirillo DM, Joosten SA, et al. Correlates of tuberculosis risk: predictive biomarkers for progression to active tuberculosis. Eur Respir J. 2016;48(6):1751-63. doi: 10.1183/13993003.01012-2016. PubMed PMID: 27836953.

2. Houben RM, Lalli M, Sumner T, Hamilton M, Pedrazzoli D, Bonsu F, et al. TIME Impact - a new user-friendly tuberculosis (TB) model to inform TB policy decisions. BMC Med. 2016;14. doi: 10.1186/s12916-016-0608-4. PubMed PMID: 27012808; PubMed Central PMCID: PMC4806495.

3. Menzies NA, Cohen T, Lin HH, Murray M, Salomon JA. Population health impact and cost-effectiveness of tuberculosis diagnosis with Xpert MTB/RIF: a dynamic simulation and economic evaluation. PLoS Med. 2012;9(11):e1001347. Epub 2012/11/28. doi: 10.1371/journal.pmed.1001347. PubMed PMID: 23185139; PubMed Central PMCID: PMC3502465.

4. Vynnycky E, Fine PEM. The natural history of tuberculosis: the implications of age-dependent risks of disease and the role of reinfection. Epidemiol Infect. 1997;119:183-201.

5. Sloot R, Schim van der Loeff MF, Kouw PM, Borgdorff MW. Risk of tuberculosis after recent exposure. A 10-year follow-up study of contacts in Amsterdam. Am J Respir Crit Care Med. 2014;190(9):1044-52. doi: 10.1164/rccm.201406-1159OC. PubMed PMID: 25265362.

6. Sutherland I, Svandova E, Radhakrishna S. The development of clinical tuberculosis following infection with tubercle bacilli. Tubercle. 1982;62(4):255-68.

7. Dye C, Garnett GP, Sleeman K, Williams BG. Prospects for worldwide tuberculosis control under the WHO DOTS strategy. Directly observed short-course therapy. Lancet. 1998;352(9144):1886-91. Epub 1998/12/24. PubMed PMID: 9863786.

8. Kunkel A, Abel Zur Wiesch P, Nathavitharana RR, Marx FM, Jenkins HE, Cohen T. Smear positivity in paediatric and adult tuberculosis: systematic review and meta-analysis. BMC Infect Dis. 2016;16:282. doi: 10.1186/s12879-016-1617-9. PubMed PMID: 27296716; PubMed Central PMCID: PMC4906576.

9. Colebunders R, Bastian I. A review of the diagnosis and treatment of smear-negative pulmonary tuberculosis. Int J Tuberc Lung Dis. 2000;4(2):97-107. PubMed PMID: 10694086.

10. Alpert PL, Munsiff SS, Gourevitch MN, Greenberg B, Klein RS. A prospective study of tuberculosis and human immunodeficiency virus infection: clinical manifestations and factors associated with survival. Clin Infect Dis. 1997;24(4):661-8. PubMed PMID: 9145741.

11. Behr MA, Warren SA, Salamon H, Hopewell PC, Ponce de Leon A, Daley CL, et al. Transmission of Mycobacterium tuberculosis from patients smear-negative for acid-fast bacilli. Lancet. 1999;353(9151):444-9. Epub 1999/02/16. PubMed PMID: 9989714.

12. Tostmann A, Kik SV, Kalisvaart NA, Sebek MM, Verver S, Boeree MJ, et al. Tuberculosis transmission by patients with smear-negative pulmonary tuberculosis in a large cohort in the Netherlands. Clin Infect Dis. 2008;47(9):1135-42. doi: 10.1086/591974. PubMed PMID: 18823268.

13. Ferebee S. Controlled chemoprophylaxis trials in tuberculosis a general review. Adv Tuberc Res. 1970;17:28-106.

14. Corbett EL, Watt CJ, Walker N, Maher D, Williams BG, Raviglione MD, et al. The growing burden of tuberculosis: global trends and interactions with the HIV epidemic. Arch Intern Med. 2003;163.

15. Tiemersma EW, van der Werf MJ, Borgdorff MW, Williams BG, Nagelkerke NJ. Natural history of tuberculosis: duration and fatality of untreated pulmonary tuberculosis in HIV negative patients: a systematic review. PLoS One. 2011;6(4):e17601. doi: 10.1371/journal.pone.0017601. PubMed PMID: 21483732; PubMed Central PMCID: PMC3070694.

16. MacPherson P, Houben RM, Glynn JR, Corbett EL, Kranzer K. Pre-treatment loss to follow-up in tuberculosis patients in low- and lower-middle-income countries and high-burden countries: a systematic review and meta-analysis. Bull World Health Organ. 2014;92(2):126-38. doi: 10.2471/BLT.13.124800. PubMed PMID: 24623906; PubMed Central PMCID: PMC3949536.

17. Churchyard GJ, Stevens WS, Mametja LD, McCarthy KM, Chihota V, Nicol MP, et al. Xpert MTB/RIF versus sputum microscopy as the initial diagnostic test for tuberculosis: a cluster-randomised trial embedded in South African roll-out of Xpert MTB/RIF. The Lancet Global health. 2015;3(8):e450-e7. doi: 10.1016/S2214-109X(15)00100-X. PubMed PMID: 26187490.

18. World Health Organisation. WHO Treatment Outcomes Database Geneva2017 [cited 2017 20th September]. Available from: <https://extranet.who.int/tme/generateCSV.asp?ds=outcomes>.

19. Stover J, McKinnon R, Winfrey B. Spectrum: a model platform for linking maternal and child survival interventions with AIDS, family planning and demographic projections. Int J Epidemiol. 2010;39 Suppl 1:i7-10. doi: 10.1093/ije/dyq016. PubMed PMID: 20348129; PubMed Central PMCID: PMC2845855.

20. World Population Prospects, the 2015 Revision [Internet]. 2015.

21. Schenzle D. An age-structred model of pre and post vaccination measles transmission. Journal of Mathematics Applied in Medicine and Biology. 1984;1.

22. Alkema L, Raftery AE, Clark SJ. Probabilistic projections of HIV prevalence using Bayesian melding. The annals of applied statistics. 2007;1(1):229-48.

23. Raftery AE, Bao L. Estiamting and projecting trends in HVI/AIDS generalized epidemics using incremental mixture importance sampling. Biometrics. 2010;66.

24. World Health Organisation. WHO Treatment Notifications Database Geneva: 2017; 2017 [cited 2017 17th September]. Available from: <https://extranet.who.int/tme/generateCSV.asp?ds=notifications>.

25. World Health Organisation. WHO TB Burden Estimates Geneva2016 [cited 2017 17th September]. Available from: <https://extranet.who.int/tme/generateCSV.asp?ds=estimates>.
